# Exploring linkages between drought and HIV treatment adherence in Africa: a systematic review

**DOI:** 10.1016/S2542-5196(22)00016-X

**Published:** 2022-04-01

**Authors:** Kingsley Stephen Orievulu, Sonja Ayeb-Karlsson, Sthembile Ngema, Kathy Baisley, Frank Tanser, Nothando Ngwenya, Janet Seeley, Willem Hanekom, Kobus Herbst, Dominic Kniveton, Collins C Iwuji

**Affiliations:** Africa Health Research Institute, KwaZulu-Natal, South Africa; Centre for Africa-China Studies, University of Johannesburg, Johannesburg, South Africa; Department of Global Health and Infection, Brighton and Sussex Medical School, University of Sussex, Brighton, UK; Department of Global Health and Infection, Brighton and Sussex Medical School, University of Sussex, Brighton, UK; United Nations University Institute for Environment and Human Security, Bonn, Germany; Institute for Risk and Disaster Reduction; School of Global Studies, University of Sussex, Brighton, UK; Africa Health Research Institute, KwaZulu-Natal, South Africa; Africa Health Research Institute, KwaZulu-Natal, South Africa; University College London, London, UK; Faculty of Epidemiology and Population Health; Africa Health Research Institute, KwaZulu-Natal, South Africa; London School of Hygiene & Tropical Medicine, London, UK; Lincoln Institute for Health, University of Lincoln, Lincoln, UK; Africa Health Research Institute, KwaZulu-Natal, South Africa; School of Nursing and Public Health, University of KwaZulu Natal, Durban, South Africa; Africa Health Research Institute, KwaZulu-Natal, South Africa; Global Health and Development Department, London School of Hygiene and Tropical Medicine, London, UK; Africa Health Research Institute, KwaZulu-Natal, South Africa; Division of Infection and Immunity, University College London, London, UK; Africa Health Research Institute, KwaZulu-Natal, South Africa; DSI-MRC South African Population Research Infrastructure Network, Durban, South Africa; School of Global Studies, University of Sussex, Brighton, UK; Africa Health Research Institute, KwaZulu-Natal, South Africa; Department of Global Health and Infection, Brighton and Sussex Medical School, University of Sussex, Brighton, UK

## Abstract

Climate change is directly and indirectly linked to human health, including through access to treatment and care. Our systematic review presents a systems understanding of the nexus between drought and antiretroviral therapy (ART) adherence in HIV-positive individuals in the African setting. Narrative synthesis of 111 studies retrieved from Web of Science, PubMed/MEDLINE, and PsycINFO suggests that livelihoods and economic conditions, comorbidities and ART regimens, human mobility, and psychobehavioural dispositions and support systems interact in complex ways in the drought–ART adherence nexus in Africa. Economic and livelihood-related challenges appear to impose the strongest impact on human interactions, actions, and systems that culminate in non-adherence. Indeed, the complex pathways identified by our systems approach emphasise the need for more integrated research approaches to understanding this phenomenon and developing interventions.

## Introduction

A recent WHO report supporting the negotiations of the UN Framework Convention on Climate Change recognised that climate change affects human health both directly and indirectly.^1^ The report notes an estimated close to 13 million deaths—about 23% of all global deaths—linked to modifiable environmental factors, often related to climate change.^2^ While direct health impacts of climate change, including physiological effects of exposure to higher temperatures and increasing incidence of non-communicable diseases, such as respiratory and cardiovascular diseases, are relatively well understood,^3–6^ indirect effects on health, particularly those resulting from long causal pathways, such as through impacts on livelihoods, are more difficult to identify.

Drought is a major consequence of anthropogenic climate change, with impacts on human health. Droughts have affected most people living in the Horn, Sahel, and southern regions of Africa with increasing frequency and duration and impact on health, exacerbated by these regions’ low adaptive capacity.^7–12^ Indeed, social vulnerabilities, especially high HIV prevalence, unemployment levels, and gender inequality further complicate the ways in which drought, and other climate and weather conditions, affect populations in Africa.^9–11,13–15^ Various studies have shown how gender roles are crucial dimensions of social vulnerability (for women and men) that can be exacerbated by drought and other environmental exposure.^10,11,14,15^

An extensive body of research links social inequality and inequity, marginalisation, and discrimination to the aggravated impacts from climatic changes or natural hazards mainly upon women due to social power relations. To give some examples, women have been reported to eat less in the event of drought-induced food insecurity; put themselves in danger to help children during floods; face sexual violence, exploitation, and abuse en route to or within cyclone evacuation shelters; often experience social punishment and financial abandonment after climate-induced male out-migration; and suffer unequal post-disaster ill-health consequences.^16^–^21^ A recent study also found that more women than men seem to commit suicide during periods of extreme heat and humidity.^17^

A study drawing upon data from 91 low-income and middle-income countries concluded that drought operates through food insecurity to heighten HIV transmission risk among vulnerable women in poor countries.^14^ Another study further investigated the role of age and gender on HIV infection. In this study, drought was associated with early sexual debut, transactional sex, and higher HIV prevalence in adolescent girls and young women aged 15–24 years in rural areas. Drought was not associated with HIV prevalence in older females (25–59 years) and was protective with respect to HIV infection in young males.^7^

In addition to pre-existing climate vulnerabilities, southern Africa in comparison to other regions in Africa has the highest HIV burden, accounting for more than 30% of the global HIV prevalence.^22^ Both direct factors (sexual behaviour and unprotected sex) and longer causal chains (lack of adherence to HIV antiretroviral therapy [ART]) contribute to this high burden.^7,8^ We focus here on the indirect impacts of drought on HIV treatment adherence in Africa because of the contributions of poor ART adherence to increased morbidity and mortality risks in HIV-positive individuals as well as HIV transmission.^7,8^

The objective of this paper is therefore to develop a systems understanding of the nexus between environmental stress, in this case drought, and ART adherence in HIV-positive individuals in Africa. Several scholars have argued for the effective use of a systems approach to draw out the many indirect linkages between climate change and health, or other outcomes, that might be difficult to attribute to the environment as the pathways pass through various social, political, economic, and psychological factors. For example, a systems understanding has previously been applied to investigate the connections between climate change and mental health,^23,24^ and natural hazards and wellbeing.^19^

The previously shown association between drought and increased HIV prevalence could be explained by changes in behaviour in reaction to income and production shocks, which often culminate in increased sexual risk taking, temporary migration, school withdrawal, and early sexual debut, especially in rural contexts.^7,8^ However, to date no study has applied a full systems approach to understand the association between drought and HIV in general, or in particular to account for the complexities underlying drought’s impact on HIV ART adherence, which in turn can affect HIV transmission. Berry and colleagues define systems thinking as a set of “synergistic analytic skills” used to help describe a complex set of interacting factors that produce outcomes, to predict their behaviour, and to formulate interventions to achieve desired results.^24^ We argue that the systems approach is more appropriate in investigating the drought–HIV nexus as it shows how different geopolitical, socioeconomic, and environmental factors, including health systems, interact through a complex interlinked process and culminate in non-linear outcomes such as mental (ill-)health, increased HIV transmission, or ART (non-)adherence, which is our focus.^23–25^ Thus, we undertook a systematic literature review on the impacts of drought on human health and livelihoods, and factors associated with ART adherence, and used the findings to develop a systems diagram describing the relationship between drought and ART adherence. This makes a case for future research agendas and policy frameworks that capture the complex causal pathways between drought and ART adherence, especially in Africa.

## Methods

Four researchers (KSO, SA-K, DK, and CCI) searched three electronic databases for peer-reviewed published literature: Web of Science, PubMed/MEDLINE, and PsycINFO (Jan 1, 2003, to Sept 20, 2019). We chose 2003 as our starting date as it was about the time ART roll-out was starting in Africa.^26^ Three distinct searches were applied to each of the databases in line with the study objective to cover publications on impacts of drought generally, impacts of drought on human health, and adherence to ART, in the African setting (panel and appendix p 2). We reviewed primary studies published in English.

We imported all articles into EndNote reference management software, version X9 (Clarivate), and excluded duplicates using the “Find Duplicates” function in EndNote. KSO, SA-K, DK, and CCI independently screened the titles and abstracts of all records to identify studies possibly related to our areas of interest. We obtained full text articles from the three distinct searches (panel) which examined (1) the general impact of drought on Africa, (2) the impact of drought on human health, and (3) HIV treatment adherence-related factors. KSO and CCI screened the full text articles based on the inclusion and exclusion criteria (panel) and CCI made final decisions on which articles to include in the review when there was a discrepancy. We included both quantitative and qualitative studies to allow us to describe both the proximal and distal factors that connect drought with HIV treatment adherence. The heterogeneity in the study designs and outcomes investigated in the included studies meant it was inappropriate to undertake a meta-analysis.

For the quality assessment of included articles, we applied the Critical Appraisal Skills Programme (CASP) quality assessment tool^27^ to specifically assess only studies linked directly to adherence as the outcome variable of interest. For quantitative and mixed methods studies, we used the CASP^27^ criteria to address the following questions: (1) is the question clear or are there clear aims and objectives? (2) Is the sample appropriate, and does the size allow generalisation? (3) Is the research design clearly stated? (4) Is the data collection process clear, including recruitment and consent? (5) Did the researcher follow the steps of data analysis and was the data management clear? (6) Are the results accurate and presented in the correct format? (7) Does the discussion and conclusion support the results?

To assess the quality of qualitative studies, a previously described adaptation of questions representing the three key conceptual domains described in the CASP quality assessment tool was used.^27,28^ The criteria addressed the following questions: (1) was the relationship between researcher and participant adequately considered? (2) Was the sampling method clearly described? (3) Was the data collected in a way that addressed the research issue? (4) Was the analysis method clearly described?

We organised the studies by year of publication, study design, country of origin, and key findings. For each search, we grouped findings into key thematic areas using NVivo 12 Pro (QSR International) and Microsoft Excel to tabulate them. Subsequently, we linked common themes across the searches to establish the relationships between drought, health, HIV, and adherence to HIV care and treatment, using the same approach for both quantitative and qualitative studies.

We followed the Preferred Reporting Items for Systematic Reviews and Meta-Analyses (PRISMA) guidelines for this systematic review. The PRISMA checklist is in the appendix (pp 36–37).

## Results

The numbers of articles derived from the search of the databases are summarised in the figure. Our search identified 3217 articles, and we excluded 503 duplicates. A further 2482 were excluded after screening abstracts for titles and relevance, leaving 232 articles relevant for full text review, of which 121 articles were excluded as they did not meet inclusion criteria on adherence as the main outcome variable of the study, situated in Africa, focused on adults, and focused on human impacts in the case of drought (panel). Studies that focused broadly on climate change without direct emphasis on drought, or those that mentioned adherence without specific focus on this outcome and related factors, were deemed insufficient and consequently excluded (figure).

After all exclusions, 111 articles were synthesised in the systematic review, including 71 quantitative studies, 27 mixed methods studies, and 13 qualitative studies (table 1). The majority of included studies were from South Africa (24), Uganda (13), Kenya (12), and Ethiopia (10). Subregionally, the majority of these studies were in southern (47) and eastern (41) Africa, with west Africa and central Africa accounting for 22 and three articles respectively. Some articles included data from more than one country across subregions; as a result, the total number of studies exceeds 111. These are summarised in two tables in the appendix (p 3).

Many studies exploring factors associated with ART adherence have relied on a socioecological framework. This framework recognises that societal norms and structures influence individual attitudes and behaviours, and identifies key levels affecting adherence to ART: individual (knowledge, attitudes, beliefs, perceptions); community (cultural values and norms); interpersonal (family, friends, social networks); institutional (health system, social institutions, workplace); and public policy (local, state, and national laws and policies).^29–31^ The most popular of these levels that we used to delineate adherence-related factors include individual, community or contextual, and health or policy systems levels (table 1). We note that of 38 adherence-related studies, 31 high-lighted individual level factors (23 quantitative, three mixed-methods, and five qualitative studies). 20 studies (13 quantitative, two mixed-methods, and five qualitative studies) addressed community and contextual level factors. Health systems factors were addressed in 11 quantitative, one mixed-methods, and two qualitative studies. All 111 studies that contributed to the synthesis are summarised in the appendix (pp 4–32). The quality assessment result showed that only five of the 31 included quantitative and mixed-methods studies were deemed to be at low risk of bias (appendix p 33) while only one of the seven included qualitative studies was found to be at low risk of bias (appendix p 34).

We used the empirically derived themes (table 2) from the quantitative and qualitative studies to develop a systems explanation of the relationship between drought and adherence (appendix p 35) while citing areas of similarity between the theoretical framework such as the socioecological model described earlier.

The four thematic areas—(1) livelihoods and economic conditions, (2) physical health constraints and ART regimens, (3) human mobility, and (4) social support and psychobehavioural dispositions (table 2)—illuminate the complex pathways to understanding the extent to which adherence to HIV care is sensitive to the effects of drought on human livelihoods and interactions, as well as structures of policy response to environmental stress.

### Livelihoods and economic conditions

Livelihoods and economic conditions emerged as one of strongest determinants of (non-)adherence to ART. Factors identified here, including food insecurity, water insecurity, and others (table 2), cumulatively affected ART adherence. Multiple studies have shown poor socioeconomic conditions to be associated with poor adherence. Often cited in the literature is the impact of worries about taking ART on an empty stomach.^32–39^ The fear of adverse side-effects linked to food insecurity was also noted as an important barrier to adherence in both quantitative and qualitative studies.^40–49^

Study participants attributed side-effects such as hallucinations, drowsiness, and sickly feelings to taking ART with insufficient food, or on an empty stomach.^39,47,49^ Drought directly affects food security through loss of production of both crop and livestock, and for subsistence farmers can affect their capacity to access food.^5,50–83^ Individuals could also be impacted indirectly either through loss of employment, or the increase in food prices.^33,57,84–87^

In response to a severe economic impact of drought, some individuals sold off assets, including those that help to meet individual and family food needs.^62,68,81,88^ In the case of an extended drought, beyond a year or growing season, this can affect mental health (anxiety, stress, depression) which in turn could impact not only individual but also family socioeconomic conditions if the breadwinner in the family is affected.^89^ ART adherence could then be affected through trading off family food provision with the cost of transportation to a health-care facility for drug pick up or vice versa.^42,47^ Conversely, individuals who are employed and well-off sometimes find themselves defaulting on treatment due to the demands of work.^33,35,36,42,45,47,90^

Lack of access to clean water, or the means to buy it, is a socioeconomic condition (table 2) also found to have severe impact on ART adherence.^37,91^ Extreme drought can further exacerbate an already limited resource. Our systems diagram (appendix p 35) shows how low water quality and quantity is linked to diseases afflicting livestock and human beings alike; for example, drought-tolerant tuber crops such as cassava can lead to Konzo disease,^64,67,70,92–94^ a type of paralysis of the leg which is permanent and is associated with the consumption of inadequately processed cassava-based food.^70^ People on ART might also forgo hospital appointments, as they search for clean water for themselves and their livestock, while insufficient water and low water quality (driven by drought) might exacerbate poor economic conditions that individuals, communities, farmers, herders, and countries face in general.^8,54,57,73,95,96^ Insufficient water impacts overall food production, increases government expenditure on food, and could exacerbate morbidity. Such fiscal burdens on the economy linked to food and water could have ripple effects on expenditure on adequate water supply to poor drought-stricken rural communities, where many HIV-positive individuals reside. This would inadvertently impact ART adherence in these communities, with poor individuals who rely on government provision of water, often inconsistent, being disproportionately affected.

Poor economic conditions, possibly from drought impact on livelihoods or overarching poverty, impede people’s ability to acquire even the simplest technological device (mobile phones), often used to facilitate ART adherence.^97^ Developing technology-based interventions— either for drought monitoring or ART adherence—heavily depends on economic viability, but in many African countries, harsh economic conditions put governments in difficult positions of making trade-offs between health interventions and economic stability, among others. Consequently, poor economic conditions and insufficient interventions interact negatively to exacerbate how drought affects the agency of individuals and communities.

The economic shocks associated with drought, especially in terms of seasonal poverty, can impact investment in human capital, including education^56,98,99^ and access to adequate health care. Previous ART adherence studies showed that HIV-positive individuals who had to bear the cost of treatment, including those linked to transportation to clinics, reported poor adherence.^42^ Various support systems,^39,45–47,100^ including community health workers, family members, lay health workers, and counselling groups within a drought-hit context might face challenges from drought that affect their ability to provide support for health care in general.

### Comorbidities and ART regimen

Drought aggravates the physical and mental health pressures that individuals face.^4–6,8,58,68,70,94,99,101^ Like other environmental stressors and extreme events, including floods, drought is linked to disease outbreaks such as Rift Valley fever, Konzo disease, trachoma, diseases linked to poor hygiene and access to water (eg, diarrhoea), and diseases carried by vectors (eg, chikungunya outbreaks in east Africa).^4–6,70^ Furthermore, drought has been linked to later life disability, especially among males who also are at risk of both physical and mental disabilities having experienced drought as infants.^101^

It is established that drought imposes economic distress and stress on individuals due to losses in crop and animal production, and livelihoods.^8,68,72^ Such stressful situations, including those linked to income loss, unemployment, seeking off-farm employment, or migration, and possibly exacerbated by drought-related diseases and disabilities, might culminate in coping mechanisms that include alcohol and substance abuse, which have been implicated in domestic and intimate partner violence (IPV).^23^ Furthermore, migration has been linked to increased risky sexual behaviours that culminate in increased prevalence of HIV in Africa.^8^

Alcohol and substance abuse cases have been shown to be associated with comorbidities like diabetes, heart diseases, hepatitis, hypertension, and strokes.^102–105^ Treatment for multimorbidity related to these conditions, in addition to acute stress and depression, results in increased pill burden, which is associated with poor adherence^6^ and increased likelihood of drug–drug interactions with HIV drugs.^43,45,46,48,49,106–108^ This could result in possible trade-off in adherence to one treatment over another. In drought, especially among the rural poor, this trade-off might be exacerbated by the lack of sufficient food and clean water further increasing disease susceptibility. Where drought and comorbidities interact with economic stress, social vulnerabilities, medication stock-out, pill burden, or side-effects from ART regimens, this can be detrimental to adherence.^39–49,109–110^

Non-adherence, a negative health-seeking behaviour, is also attributable to patients’ experience of, and relationship with, health-care providers.^39,41,47,90^ Three studies cited the attitudes of nurses towards patients as a possible barrier to ART adherence, including not trusting health facilities to maintain confidentiality.^34,45,47^ This could be an additional disincentive in patients already facing increased livelihood challenges from drought, especially among men who, facing a dominant masculine normativity of breadwinner, feel ashamed, isolated, and refuse to seek help for their mental illness due to the stigma attached to mental health.^23^ Drought’s impact on the macro-economy might arguably also exacerbate drug stock-out of more expensive ART regimens with fewer side-effects, as countries might rely on cheaper, older regimens with more side-effects due to competing policy priorities.

### Human mobility

Human mobility, with mobility defined broadly to encapsulate migratory activities as well as other forms of movements, brings together factors that can influence adherence. Many studies showed that adherence is very sensitive to mobility, as people sometimes move from where they are resident and registered with health-care facilities to new and possibly unfamiliar places.^46,91,108,111^ Some included studies showed that drought is a very strong driver of human migration, with people moving away from drought-affected areas or relocating their livestock to areas where they could find forage and water to prevent the livestock from dying.^7,8,71,95,112–116^ The impact of drought on adherence could be mediated through this forced mobility.

Collectively, these studies show how forced migration from drought or other forms of migration can affect adherence. For example, permanent migration (change of residence)—where people changed locations and lost touch with their primary clinics—or travels outside normal areas of residence for work (including holidays or religious activities of some sort) affected adherence among patients.^91,111^ Similarly, movements generally linked to seeking off-farm employment or permanent relocation out of a drought-stricken area were found in relation to the impact of drought on livelihood.^62,75,117,118^

Drought has been linked to violence and displacements as well as increased risky sexual behaviour and alcohol and substance abuse, which have been shown to be associated with poor adherence.^8^ Firstly, the scramble over water sources by herders and farmers is well recorded in countries like Kenya and Nigeria.^118,119^ Although the impact of drought has not been explored in depth in the Nigerian case, in Kenya, incidents of violence linked to the practice of cattle-rustling emanate from scarce water resources.^115,119–121^ In contexts where such violence leads to large-scale displacement through forced migration, the implication for HIV-positive individuals in care becomes dire. Secondly, people who migrate in search of better life opportunities out of drought-stricken areas face uncertainties in their destinations that have culminated in many risky sexual behaviours such as transactional sex and alcohol abuse, both of which are strongly associated with poor adherence.^7,8^

Droughts cause crop failures, production losses, livestock deaths or reduced productivity, and almost total destruction of individual and collective livelihoods to the extent that social structures (social networks) within socially knit populations become stretched.^55^ People move, temporarily or permanently, internally or internationally, to seek out avenues to survive. We found these to include taking refuge outside the drought area, sending children to more affluent relatives (maybe outside the area), sending family members abroad,^55,71,114,122^ selling off their assets to survive, abandoning rural farms to seek off-farm employment in cities, or even, for cattle farmers, moving their herd away in search of forage in other towns or areas.^55,62,64,75,87,112,113,122^ These outcomes linked to drought, as our review found, are some of the drivers of poor adherence.

Consequently, the mitigation strategies established to manage drought impacts were individual-based, such as moving or sending children or family members to relatives or abroad, community-focused, such as food or loan support systems, and even on an institutional or policy level of intervention.^55,79,95,122–124^ An important issue about the latter is that if, and once, drought succeeds in destabilising support systems set up individually or collectively—within societies and beyond—then the devastation on wealth and health further diminishes resilience.^55,122^ This is central to the next theme; support systems and the linkages to drought and adherence.

### Social support and psychobehavioural disposition

Our review has shown that migration can mean the loss of important support structures, especially strongly knit (society or family) social support systems. This could be a source of anxiety and stress relating to concerns around adjustment and integration in the new environment.^23^ The place and role of support systems for adherence is well documented in the ART adherence literature. In fact, issues linked to support systems were described in 17 articles highlighting how marital status, non-disclosure, and forgetfulness drive poor adherence, and how caregivers’ roles, counselling groups, and community or lay health workers were crucial towards facilitating adherence.^34,35,42,44,47–49,91,100,106,107,125–131^ Conversely, failures in the support system, such as absence of caregivers or lack of health workers, were shown to be detrimental to ART adherence in different population groups, with younger age groups being more affected.^43,44,84,129^

The sensitivity of adherence to the support systems appeared to be exacerbated by gender, which is similarly impacted by drought.^34,56,72,108^ Indeed, where support systems are lacking, livelihood losses induce stress that leads to alcohol and substance abuse and associated risky sexual behaviour, including transactional sex, multiple sexual partners, and sex without condom use, due to weak bargaining power (for women and girls), which studies showed to hinder adherence.^7,32,43,45,47,89,132^ Very importantly, these behaviours can emanate from attempts to cope with HIV-related stigma and unpalatable experiences from health workers.^34,39,41,49,90^ Some of these negative coping strategies might inadvertently lead to domestic violence (including IPV), which in some cases also negatively affects adherence.^109,127^

Social, cultural, and religious norms that normalise such systems of stigmatisation, especially in paternalistic African societies, exacerbate this situation.^34^ Drought, by increasing vulnerability within affected populations, constrains (or possibly erodes) whatever safety nets and support structures that might exist.^55,95,122^ It imposes additional shocks in a situation where poverty already disrupts individuals’ capacity to support themselves or extended family members who might be dependent on them for sustenance.

## Discussion

The individual and public health consequences of poor ART adherence, and resulting increase in HIV drug resistance, have been clearly described.^133^ At the individual level, these consequences include increased HIV-related morbidity and mortality, while at the public health level, there is the risk of transmission of, possibly drug-resistant, HIV to sexual partners, and a threat to national HIV treatment programmes based on the public health approach as in many African countries.^134^

In this systematic review, we utilised a systems approach to examine the complex linkages between drought and ART adherence, which are mediated through a web of proximate and distal factors not often considered. We found that the strongest links between drought and poor ART adherence were those clustered around livelihoods and economic conditions, with most emphasis on food insecurity, loss of production, and individual, societal, and national economic conditions in the form of unemployment and reduced overall income. These factors interact with social support systems, psychobehavioural dispositions, mobility, physical health,^135^ and ART regimen-related constraints in a disruptive manner, culminating in varying forms of poor adherence.^23^

Our systems diagram (appendix p 35) connects these factors, demonstrating adherence sensitivity to drought-related impacts. So, elements of poor adherence, such as medication side-effects, comorbidities, and migration, are shown to be largely products of constrained livelihood and economic conditions. Such conditions have also been shown to exacerbate stress, depression, stigma, alcohol and substance misuse, risky sexual behaviour, and IPV.^23,136^ Consequently, non-disclosure or forgetfulness, and inadequate support structures, prove detrimental to adherence. IPV was not shown to be associated with poor adherence among HIV-positive sex workers^127^ in one included study, while in another,^109^ IPV was associated with treatment interruption in women living with HIV. A higher report of commercial and forced sex was observed in drought-affected areas in Lesotho than in non-affected areas, attributable to household poverty. Hence, drought can impact adherence by resulting in increased violence towards women, regardless of involvement in sex work.^7^

The reviewed literature described individual, societal, and institutional strategies to mitigate economic situations and address poor adherence. Institutional and policy frameworks related to drought and environmental stress mitigation include water harvesting, collective loan systems, food aid, drought tolerant crops, off-farm employment, or alternative livelihoods, among others.^61,62,64,66,68,74,77,83,87,88,117,118,122–124,137–144^ The review also shows the disruptive effects of drought on these strategies.^55,122^

Adherence studies allude to policy and health system factors and their impact on adherence. Statistical evidence (and good qualitative narratives from some of these studies) of associations between health systems interventions and positive adherence outcomes among patients have been found in quantitative studies.^38,100^ This means that changes in medication (ART) regimens, improved policy guidelines for patients’ handling or engagement (targeted at care providers and facilities), sentiments of trust towards care providers, and the effectiveness of counselling and support groups and community or lay health workers signalled improvements in adherence outcomes in the reviewed literature. This highlights the essential role of policy and health systems as regards these positive outcomes. Conversely, failure within these systems is detrimental to adherence.

Noteworthy, and linked to drought impact mitigation, is that poor economic conditions—a possible impact of drought—can detract from institutional frameworks’ attempts to address the challenges of livelihood losses and non-adherence. Because the effects of drought strike deep into the economy, it could raise the country’s debts and increase the opportunity costs that might truncate strides to cushion both environmental stress and adherence challenges.^23^ A strong and resilient economy is more favourable towards advancing ART adherence and limiting its negative consequences by ensuring sufficient medication supplies and allowing review and reform of medication regimens with increased adverse effects on patients, while also enhancing and supporting food supply systems and various networks of support for HIV-positive individuals.

Our review had some limitations. Prominent among them is that we did not include grey literature and studies not published in English. Although some of these pieces of literature might have been relevant for this review, especially those not published in English, we omitted them because of the challenges of translating non-English published sources. Also, due to the paucity of data linking drought and HIV ART adherence, discussions pertaining to possible experiences of adolescent girls and young women, who are disproportionately affected by HIV in the African setting, were limited to the impact of drought on HIV prevalence,^145,146^ while other key populations such as men who have sex with men, and climate-induced gender impacts upon men, were largely not explored in the studies included in this review.

This review, bringing together environmental and physical related factors linked to drought and various barriers to and facilitators of ART adherence in Africa, demonstrates the strength of a systems approach. The triangulation of quantitative, qualitative, and mixed-methods studies enhanced the ability of this study to elucidate complex connections between drought and adherence that were not immediately apparent. This is crucial for future studies on the interaction between drought and HIV-related treatment and adherence challenges.

### Gaps in literature: towards further research

One major finding from this systematic review is the lack of studies directly investigating the relationship between drought and ART adherence. The systems approach adopted by our review substantially extends the literature in this field by exploring the non-linear and complex pathways between drought and HIV treatment adherence.

The cross-sectional nature of the two studies that examined the relationship between drought and HIV prevalence meant that the association could not be considered causal, as acknowledged by the authors.^7,8^ Furthermore, HIV prevalence is a weak outcome variable because it is sensitive to the mortality rate and HIV incidence in the population, which in turn are affected by factors other than ART adherence per se. Longitudinal studies investigating the impact of drought on HIV acquisition, and mediators such as population HIV viral load, could therefore address the noted shortcomings.

Furthermore, mental health challenges (acute stress, anxiety, depression, trauma), as well as stigma, are crucial to understanding the drought–adherence nexus, since these challenges can exacerbate economic hardship and vice versa.^39,90,100,132,135,147^ Surprisingly, only one article focused on the impact of drought on mental health in Africa, even though stress, broadly speaking, can provide a convergence point in grasping drought’s impact on ART adherence.^89^ The dearth of information in this area highlights the fact that mental health is under-investigated in the African setting, especially in the context of medical pluralism, in which mental health conditions are often attributed to spiritual or ancestral issues. This is an area that warrants further investigation. There was also a paucity of studies investigating the impact of drought on key populations, and the systems approach we have used to describe the drought–adherence nexus might not apply to these groups, thereby highlighting future areas of investigation. Studies on appropriate mitigation strategies and economic support systems for less resilient economies with a high burden of HIV and individual poverty with limited ability to cushion the impact of drought are urgently required.

## Supplementary Material

Appendix

## Figures and Tables

**Figure:**
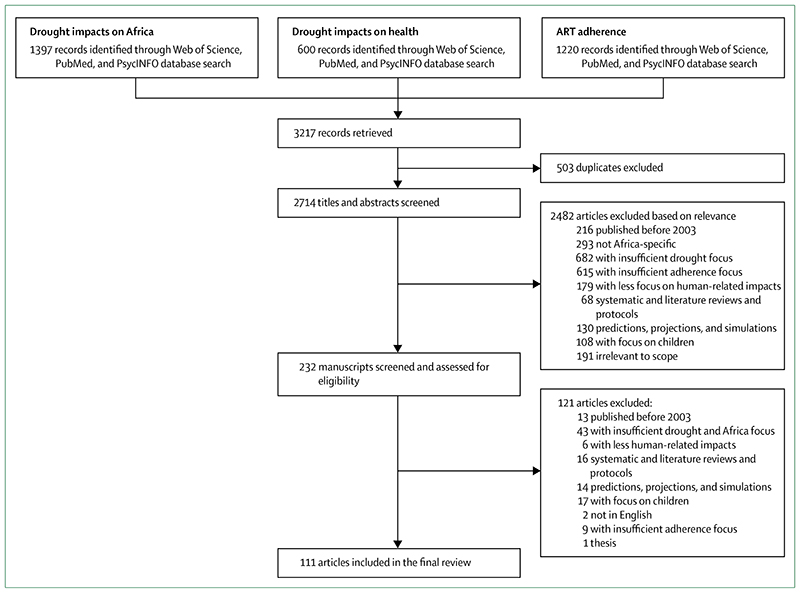
Flow chart of search results and included studies ART=antiretroviral therapy.

**Table 1:** Reviewed studies by methods and socioecological level

	Quantitative studies	Mixed methods	Qualitative studies	Total
All reviewed studies (n=111)				
Drought impacts on Africa	33	22	5	60
Drought impacts on health	9	3	1	13
ART adherence	29	2	7	38
Total	71	27	13	111
Reviewed adherence-related studies (n=38)				
Individual level	23	2	6	31
Community or contextual level	13	2	5	20
Health system or policy level	11	1	2	14
Total	47	5	13	65

ART=antiretroviral therapy.

**Table 2:** Thematic areas showing interlinked factors between drought and ART (non-)adherence

	n (%) studies
Livelihoods and economic conditions (total studies=76)	
Catastrophic treatment costs	2 (3%)
Drought impact mitigation	23 (30%)
Food insecurity	22 (29%)
Poor ART knowledge	4 (5%)
Intervention programmes	2 (3%)
Loss of production	20 (26%)
Low water quality and quantity	5 (7%)
Missing education	4 (5%)
Poor individual and national economic conditions	5 (7%)
Reduced livelihood diversification	2 (3%)
Selling off assets and borrowing	6 (8%)
Technology	1 (1%)
Unemployment	15 (20%)
Social support and psychobehavioural disposition (37)	
Age	4 (11%)
Alcohol and substance abuse	6 (16%)
Fertility choice	6 (16%)
Inadequate counselling or support groups	6 (16%)
Depression and mental ill-health	5 (14%)
Forgetfulness	3 (8%)
Gender	4 (11%)
Lack of CHWs and LHWs	1 (3%)
Less equitable gender norms	2 (5%)
Marital status	2 (5%)
Non-disclosure	7 (19%)
Risky sexual behaviour	2 (5%)
Stigma	6 (16%)
Traditional belief and treatment	2 (5%)
Violence	8 (22%)
Comorbidities and ART regimens (27)	
Medication side-effects	11 (41%)
Nurses’ and officials’ behaviour	4 (15%)
Drought-related diseases	2 (7%)
Comorbid conditions	16 (59%)
Stock-out	2 (7%)
Time on ART or fatigue	4 (15%)
Human mobility (17)	
Migration and displacement	14 (82%)
Seeking off-farm employment	2 (12%)
Travel away from home but not migration	3 (18%)

The numbers shown in the bold row headings do not represent the cumulative numbers from individual factors, but the unique studies linked to each factor. ART=antiretroviral therapy. CHWs=community health workers. LHWs=lay health workers.

## Key messages

- Climate change affects human health and livelihoods in a myriad of ways
- Some of the impact of climate change on human health can be through indirect mechanisms, such as the relationship between drought and HIV treatment adherence
- A systems approach allows an understanding of these indirect relationships by elucidating the distal and proximate connections between drought and HIV treatment adherence
- Our systematic review suggests that during droughts, livelihoods and economic conditions, comorbidities and antiretroviral therapy regimens, human mobility, and psychobehavioural dispositions and support systems interact in complex ways that could influence HIV treatment adherence
- Economic and livelihood-related challenges resulting from drought-induced food and water insecurity appear to impose the biggest threat to HIV treatment adherence
- Interventions to improve the health and wellbeing of people living with HIV should not just focus primarily on HIV but should adopt a whole systems approach to improving public health in general, such as ensuring water and food security

## Panel Search strategy and inclusion and exclusion criteria

### Search strategy

- Impact of drought: “Drought” AND “impact” AND “Africa”
- Drought and health: “Drought” AND “health” AND “Africa”
- ART adherence: (“HIV” or “Human Immunodeficiency Virus” or “AIDS” or “Acquired Immuno-Deficiency Syndrome”) AND (“antiretroviral” or “ART” or “ARV”) AND (“viral load” or “RNA”) AND (“adherence” or “compliance” or “non-adherence” or “noncompliance” or “treatment adherence” or “treatment compliance”) AND “Africa”

### Inclusion and exclusion criteria

- Inclusion criteria: HIV positive; 2003–19; rural or urban; food insecurity; drought; Africa; environmental migration; ART; adherence; viral load; human impacts (socioecological)
- Exclusion criteria: HIV negative; before 2003; non-environmentally affected households; broad climate change focus and future predictions; beyond Africa; children; thesis; non-English publications; non-socioecological impacts
