# Supplementary material for: Exploring linkages between drought and HIV treatment adherence in Africa: a systematic review
Source: Lancet Planet Health. Author manuscript; Available in PMC 2022 Jun 30. (PMC7612934; doi:10.1016/S2542-5196(22)00016-X)
Supplement: Appendix [file EMS146239-supplement-Appendix.pdf]

THE LANCET
Planetary Health

Supplementary appendix

This appendix formed part of the original submission and has been peer reviewed. We post it as supplied by the authors.

Supplement to: Stephen Orievulu KS, Ayeb-Karlsson S, Ngema S, et al. Exploring linkages between drought and HIV treatment adherence in Africa: a systematic review. *Lancet Planet Health* 2022; **6:** e359–70.

Web Appendix

List of investigators:

Kingsley Stephen Orievulu, Sonja Ayeb-Karlsson, Sthembile Ngema, Kathy Baisley, Frank Tanser, Nothando Ngwenya, Janet Seeley, Willem Hanekom, Kobus Herbst, Dominic Kniveton, Collins C Iwuji

Africa Health Research Institute, KwaZulu-Natal, South Africa (KS Orievulu PhD, S Ngema PGDip, K Baisley MSc, Prof F Tanser PhD, N Ngwenya PhD, Prof J Seeley PhD, Prof W Hanekom MBChB, K Herbst MSc, CC Iwuji MD);

Centre for Africa-China Studies, University of Johannesburg, Johannesburg, South Africa (KS Orievulu PhD)

Department of Global Health & Infection, Brighton and Sussex Medical School, University of Sussex, Falmer, Brighton BN1 9PX, United Kingdom (KS Orievulu PhD, CC Iwuji MD, S Ayeb-Karlsson PhD)

United Nations University Institute for Environment and Human Security, Bonn, Germany (S Ayeb-Karlsson PhD)

Institute for Risk and Disaster Reduction, University College London, London, United Kingdom (S Ayeb-Karlsson PhD)

Faculty of Epidemiology and Population Health, London School of Hygiene and Tropical Medicine, London, United Kingdom (K Baisley MSc)

Lincoln Institute for Health, University of Lincoln, Lincoln, LN6 7TS, United Kingdom (Prof F Tanser PhD)

School of Nursing and Public Health, University of KwaZulu Natal, South Africa (N Ngwenya PhD)

Global Health and Development Department, London School of Hygiene and Tropical Medicine, London, United Kingdom (Prof J Seeley PhD)

Division of Infection and Immunity, University College London, London, United Kingdom (Prof W Hanekom MBChB)

DSI-MRC South African Population Research Infrastructure Network, Durban, South Africa (K Herbst MSc)

School of Global Studies, University of Sussex, Brighton, United Kingdom (Prof D Kniveton PhD, S Ayeb-Karlsson PhD).

Corresponding to:

- Dr Collins C Iwuji
- Department of Global Health & Infection,
  Brighton and Sussex Medical School, University of Sussex,
  Falmer, Brighton BN1 9PX, United Kingdom
- c.iwuji@bsms.ac.uk

Appendix I: PICO Table

P<br>population<br>problem	I<br>Interest	C<br>Context	O<br>Outcome
**HIV+**	Drug-resistance in relation to HIV	HIV+ people and drug resistance in (South) Africa linked to drought	Drought, migration and interrupted care
**All ages**	ART		Drought, food insecurity and interrupted care
**Male/Female**	ARV		Drought, education and interrupted care
**Adherence**	Adherence		Drought, socio-ecological pressure, risky behaviour, interrupted care
**Drought, health and medical care**	Food insecurity		Drought, mental/physical ill-health, interrupted care
**Africa**	Social (power dynamics) / Cultural limitations to treatment		Drought, poverty, cognitive impairment, interrupted care
	Environmental migration and medical care		
	Resource conflict		

Appendix II: Table of number of studies by countries

	Study Location	No. of Studies	Sub-region
**1**	Eswatini	2	Southern Africa
**2**	Benin	3	West Africa
**3**	Botswana	2	Southern Africa
**4**	Burkina Faso	7	West Africa
**5**	Cameroun	2	Central Africa
**6**	DRC	1	Central Africa
**7**	Ethiopia	10	East Africa
**8**	Ghana	6	West Africa
**9**	Kenya	12	East Africa
**10**	Lesotho	1	Southern Africa
**11**	Mali	1	West Africa
**12**	Malawi	4	Southern Africa
**13**	Mozambique	2	Southern Africa
**14**	Nigeria	4	West Africa
**15**	Rwanda	1	East Africa
**16**	Senegal	1	West Africa
**17**	South Africa	24	Southern Africa
**18**	Tanzania	5	East Africa
**19**	Uganda	13	East Africa
**20**	Zambia	7	Southern Africa
**21**	Zimbabwe	5	Southern Africa
**22**	Africa-wide (broad area)	6	
**23**	Broad area (East Africa)	1	
**24**	Broad area (sub-Saharan Africa)	4	
**24**	Broad area (East and Southern Africa)	1	

Appendix III: Table of number of studies by sub-Saharan Africa (sub-regions)

	Study Location	No. of Studies
**1**	Southern Africa	47
**2**	Eastern Africa	41
**3**	Western Africa	22
**4**	Central Africa	3
**5**	Broad area (Africa-wide)	6
**6**	Broad area (East Africa)	1
**7**	Broad area (sub-Saharan Africa)	4
**8**	Broad area (East and Southern Africa)	1

Appendix IV: Summary of publications meeting inclusion criteria

	Drought impact in Africa						
	**Author(s) + Year**	**Study design / Data collection**	**Sample characteristics**	**Country/Study setting**	**Study objectives**	**Main determinants**	**Study Finding(s) – Summary**
1	Anyamba A et al 2014.[5]	Quantitative study: Geo-referenced environmental and weather data.	Unclear	East Africa embedded within Global analysis.	Track significant worldwide weather anomalies that affected agriculture and vector-borne disease outbreaks (2010–2012).	Extreme weather (Rainfall, temperature change and drought)	Droughts (floods) caused reduced crop yields and the outbreak of vector-borne diseases like Dengue, Rift Valley fever, Murray Valley Encephalitis and West Nile Virus disease, especially in East Africa's 2010/11 experience.
2	Anyamba A et al 2012.[6]	Quantitative (cross-national) study: Baseline case-location data from human and livestock epidemiological surveys on outbreaks of Rift Valley fever in East Africa, Sudan, and Southern Africa.	Unclear	East and Southern Africa embedded in Global analyses.	Determine the ecological and climatic conditions leading to and associated with Rift Valley fever and chikungunya mosquito borne disease outbreaks.	Rainfall variability, Drought	Chikungunya outbreaks were attuned to abnormally high temperatures and drought, especially in East Africa. The effects of these on livestock and human beings are obvious. Vaccinating livestock was crucial to reduce losses and illness, even for human beings. But where possible, migrating - even temporarily - may be an option too.
3	Low AJ et al 2019.[7]	Quantitative study: data from Population-based HIV Impact Assessment (LePHIA) conducted after the 2014-2016 drought in Lesotho. Random sampling used to identify individuals.	Study population (n=12 887) consisted of individuals aged 15-59. 12 052 of all participants lived in the drought-affected areas (7281 in rural while 4771 were urban)	Lesotho: Nation-wide coverage (urban/rural)	Assess whether people living in areas most severely affected by the drought had higher HIV prevalence or changes in risk behaviours; and whether there was any difference in the continuum of care among PLHIV.	Drought	Drought in Lesotho was associated with higher HIV prevalence in girls 15–19yrs in rural areas, and lower educational attainment and riskier sexual behaviour in rural females 15–24 yrs. Migration was associated with 2-fold higher odds of HIV infection in young people.
4	Lobell et al 2011.[50]	Quantitative study: Empirical (simulation) trial using Fixed Effect model	17 713 trials with optimal management and 3 244 with managed drought stress for the period 1999-2007.	Africa-wide (No specific context)	To estimate climate impacts on crop yields particularly in tropical regions	Droughts and precipitation	Final maize yield reduced by 1·7 percent and drought and high temperature-prone African contexts face declines in production yield due to drought conditions amongst others.
5	Lunde and Lindtjorn 2013.[51]	Quantitative study: reconstructed cattle density and distribution over two-periods (1955-1960; 2000-05).	Unclear: Study data focused on national cattle holdings linked to spatial data with country level estimates represented in geo-referenced map points	Africa-wide	Quantify and assess the effect of climate variability on national cattle holdings in Africa from 1961-2008	Temperature, Precipitation and conflicts	Incremental global warming has seen declines in cattle population as observed in Northern Somalia, Northern Kenya, around the Niger river, Mauritania, parts of South Africa, Mozambique, Namibia, and Madagascar. Although there are large variations, dry areas have seen more reductions than wetter areas, which saw an increase.
6	Bartzke et al 2018.[52]	Quantitative study using rainfall data from 15 rain gauges from 2 timeframes	Severe drought year = annual or seasonal rainfall below the estimated 10-year return level (Less than 0·1	Narok Town, Kenya	Quantify trends and variation in rainfall in the Maasai Mara ecosystem in East Africa to grasp the implications for	Severe Drought and increased flooding	Severe Drought and increased flooding have effects on animal population dynamics, resource scarcity and possible migration patterns

		(1965-2015 and 1913-2015) in Kenya.	rainfall probability). Severe Flood years = annual rainfall above the 10-year extreme return level.		animal population and biodiversity dynamics.		
7	Angassa & Oba 2007.[53]	Quantitative study using long-term cattle population data from randomly sampled households from peasant associations (PAs). Additional: semi-structured interview with herders.	n=6 PAs; and n=72 households' responses (n=36 households from the Tula well and Pond-water rangelands) from both Dirre and Yabello districts	Ethiopia: Yabello and Dirre districts.	Analyse the relationship between long-term rainfall and cattle population dynamics. The impact of multiple droughts on calving rates and herds die-offs.	Rainfall variability, Drought	Greater reductions in calving rates during droughts implied reduced herd growth potential. Breeding females and immature animals were influenced to a much greater degree by inter-annual rainfall variability than were mature males. The data showed a downward spiral for the total cattle holdings over a 21-year period, with a decline of 54%.
8	Call et al 2019.[54]	Quantitative study: Gridded climate-household survey linked data.	n=120 communities, n=850 households, and n=2000 agricultural plots for the years 2003 and 2013.	Uganda	Explore the impact of climate anomalies on farmers' on and off-farm livelihood strategies.	Extreme climate events	Droughts decrease agricultural productivity in the short term and reduce individual livelihood diversification in the long term. Smallholder farmers cope with higher temperatures in the short term, but in the long run struggle to adapt to above-average temperatures, which lower agricultural productivity and reduce opportunities for diversification.
9	Hassan A G et al., 2019.[55]	Qualitative design: FGDs with farmers and government expert officials from the Yobe state Ministry of Environment	2 FGDs focused on farmers (19) and government officials (6). The total study population was 25.	Nigeria: Yobe state	To investigate socioeconomic impacts of drought in Yobe state, and to proffer mitigation recommendations for the state	Drought	Drought impacts were felt in reduced harvest, damaged crops, lost harvest, and livestock mortality. Some of the coping strategies local to farmers such as traditional grain loans (to be repaid at harvest without interest) and storage of excess from good harvest times were noted to have been rendered non-existent/ineffective since drought decimated farmers to a point that they could not assist one another.
10	Hyland and Russ 2019.[56]	Quantitative (cross-national) study spanning across 19 Sub-Saharan African countries. Combined household survey, Demographic Health Survey, and gridded climate data.	Analysis focused on 73 percent of women (emphasis on non-migrating women (n=76, 914)) who were living in rural areas.	Sub-Saharan Africa: Women living in rural areas in the countries studied.	Examine long-term impacts of drought exposure on women born in 19 Sub-Saharan African countries across 4 decades	Drought, Average Monthly temperature	Women exposed to extreme drought earlier on in their childhood, and raised in rural households, were (found to be significantly) less wealthy as Adults. They also had fewer years of formal education as well as reduced Adult Height. Finally, their offspring also tend to be born with low birth weights.
11	Kilimani et al., 2018.[57]	Quantitative design: Computational Generalised Equilibrium modelling - Econometric approach – focused on the Uganda Applied	The UgAGE database is made up of n=37 industries and commodities, including 25 within the broader agriculture sector, and the	Uganda: Country-level analysis	Investigate how a drought which initially affects agricultural productivity can ultimately affect an entire economy.	Drought and loss of production	Econometric analysis show that Drought causes GDP to decline because it directly lowers productivity across various agriculture industries, thereby reducing the level of agricultural output,

		General Equilibrium (UgAGE) theory and database.	data elaborates demand and supply of taxable water in the economy.				which then leads to a temporary shutdown of capital in downstream manufacturing industries; drought also impacted household consumption, causing it to decline. On the whole, drought shocks negatively affected real GDP, industry output, employment, the trade balance and household consumption.
12	Yiran, G. A. B. and L. C. Stringer, 2016.[58]	Quantitative spatial, time-series analysis	Unclear	Ghana: The savannah ecosystem	Understand the nature of hazards (their frequency, magnitude and duration) and how they cumulatively affect humans	Temperature and windstorms were analysed from the observed weather data.	Impacts from each hazard varied spatio-temporally. Within the study period, more 70% of years recorded severe crop losses with greater impacts when droughts and floods occur in the same year, especially in low lying areas. The effects of crop losses were higher in districts with no/little irrigation
13	Mare, F., et al., 2018.[59]	Quantitative study: Primary data from commercial livestock producers. Multi-staged and simple random sampling techniques used to select provinces and commercial livestock producers.<br>Additional data:<br>Open-ended and surveys.	Study population (n=350) consists mainly of commercial livestock producers from 7 out of 9 provinces in South Africa	South Africa: 7 Provinces (Gauteng and Mpumalanga excluded).	Assess the impact of the 2015 (agricultural) drought on commercial livestock producers in South Africa, with special reference to the associated drought adaptation strategies.	Drought	From 2014 to 2015, provinces in South Africa recorded a reduction in their herd numbers as a result of drought. The largest reduction was noted from 2015 to 2016 due to the devastating influence of the very dry year experienced in 2015. On average, Drought significantly affected herd size, livestock feeding and sheep flock. Government failed to assist Livestock producers and most farmers lacked preventive measures in place during the drought.
14	Shi, W. J. and F. L. Tao, 2014.[60]	Quantitative (longitudinal) study: Data drawn from climate and maize production indexes for each country in Africa	Maize yield data covering 1961-2010 for 52 African countries; mean historical temperature and precipitation for the same range (climate data); and geo-spatial (gridded irrigation data)	Africa-wide study Cross-national study	Assess the vulnerability of African maize yields to climate change and variability with different levels of management at country scale between 1961 and 2010.	Temperature change and Precipitation	Maize was the crop with the largest area in Africa in 1961–2010, but many African countries had lower maize yields (less than 1,500 kg/ha) during these years. During the maize growing seasons 1961-2010, each 1 degrees C of T-mean increase resulted in yield losses of over 10% in eight countries and 5-10% in 10 countries, but yields increased by more than 5% in four relatively cool countries.
15	Yengoh, G. T. 2013.[61]	Quantitative (explorative) study using climate and crop production data for 20 years in Cameroon's Sudan-Sahel region	Unclear	Cameroon: The Sudan-Sahel region	Find patterns of correlation on how influential rainfall is on agricultural production in the Sudan-Sahel of Africa	Rainfall and proactive policies that support food production	The influence of rainfall in agriculture can be diluted by proactive policies that support food production. Proactive policies also reduce the impact of agriculturally relevant climatic shocks, such as droughts on food crop yields over the time-series

16	Clarke et al., 2012.[62]	Mixed methods: Questionnaire/interviews on climate change perceptions. Stratified random sampling (using GIS) was used to select farms from where participants were selected.	N=18 farmers interviewed; ages ranged from 33 to 63 years (average 47 years). n=16 farmers had secondary or tertiary education, with n=2 farmers having only a primary education.	South Africa: The Great Fish River Valley, situated btw Grahamstown and Alice, within the Makana Local Municipality of the Cacadu District Municipality.	Combine the areas of climate change threats and impacts, farmers' drought coping strategies and carbon farming to understand how commercial game and livestock farmers in the Great Fish River Valley might respond and adapt to future climate change.	Extreme climate (drought) events	Due to the 2009/2010 Drought, farmers and livestock owners involved livestock and game farming faced: i) increased input cost and reduced birth rates by (live) stock, as well as grazing shortages; ii) decreased farm revenue; increase land degradation and animal deaths; iii) reduced forage availability in farms as grasses could not recover due to erratic rainfall and low water capacity to fill the dam, thus negatively impacting irrigation; and iv) increased incidents of pests and diseases. Coping strategies adopted included: reducing livestock numbers (for example by selling some of them); diversifying livelihoods where possible (some also engaging in tourism to complement farming).
17	Codjoe & Owusu, 2011.[63]	Mixed methods: Structured and open-ended questionnaire. Participants' selection relied on simple random sampling.	Twelve (n=12) households were surveyed. Four (n=4) households were randomly selected from each of the three wealth categories in the three study communities totalling (n=36) households.	Ghana: Three communities in the Afram plains (Xedzodzoekope, Mim Kyemfere and Boakyekrom).	The study examines the impact of climate change and variability on food systems	Extreme climatic events including drought	Extreme climate events affected rural food production, transportation and processing as well as storage.
18	Cooper & Wheeler, 2017.[64]	Mixed methods: Semi-structured, guided interviews; Stratified random sampling; and participatory techniques. Results triangulated with Qualitative data from FGDs and key informant interviews (KIIs).	Study population (n=160 farmers) interviewed.	Uganda: Mbarara district, South-West Uganda	Evaluate the vulnerability of rural subsistence farmers in Uganda to climate risk	Drought, extreme rainfall, soil infertility, pest, diseases, decreasing income as a result of economic instability, increased soil erosion, land shortages and falling market prices.	Drought posed a major risk for farmers categorised as wealthier while extreme heavy rainfall was perceived to be the major risk among the poorer farmers. Risks associated with drought included: loss of livestock through death and diseases; reduced income as a result of losses; crop failure; reduced water quality and poor pasture; increased malaria, less energy and flu.
19	Derbile et al., 2016.[65]	Mixed methods: Survey and In-depth (IDIs) data from three randomly selected communities in Ghana.	IDIs (n=150) households; n=15 FGDs; and Survey population (n=540) randomly sampled households in 18 communities across six districts.	Ghana: Upper West Region.	Assess the vulnerability of smallholder agriculture to climate variability, particularly incidences of drought and heavy precipitation in Ghana.	Drought and Floods	Smallholder agriculture is significantly vulnerable to climate variability especially in the context of droughts and heavy precipitation that adversely affected farmers' crops and livestock. Consequently, farmers' adaptation strategies (to mitigate these risks) come with associated risks including total livelihoods failure and food insecurity emanating from climate variability.

20	Mthembu & Zwane, 2017.[66]	Mixed methods: FGDs, IDIs and survey data	n=22 households in the Ncunjane community; household sizes range from 4 to 21 members with adult ages ranging between 18 and 93 years. In total, (n=204 sample population): 64 (59·8%) women and 43 (40·2%) men from a total of 107 adults in the population sample of 204	South Africa (Msinga, KwaZulu-Natal)	Investigate the adaptive capacity of the Ncunjane farming community in Msinga, KwaZulu-Natal in response to drought spells of 2010 and 2014	Variability of rainfall and prolonged heat spells, Cattle ownership	Both Livestock and crop farmers faced high cattle mortalities and stagnant crop productivity that pressurized their constrained disposable household income because of increased food costs and agricultural input costs, particularly supplementary animal feed. Cattle owners were more vulnerable to drought because of poor risk management and thus became highly dependent on government to provide drought relief.
21	Terry, A. K., 2020.[67]	Mixed methods: data collection through surveys of randomly sampled participants from three farmers' associations of the KDDP which adopted irrigated home gardens.<br>Additional data: IDIs with community members of the associations.	n=60 households participated in the survey questionnaire consisting of n=17 (members of Bhelebesevi Multi-purpose Co-operative), n=19 (Sivukele) and n=24 (Intamakuphila). These make up 21 per cent of the combined membership, but approximately 50% of those with irrigated home gardens.	Swaziland	Explore the impact of the 2015-16 El Nino drought on irrigated home gardens on the Komati Downstream Development Project in Swaziland.	Drought	Results showed that between November 2015 and April 2016 maize production failed; 93% of irrigated gardens produced no maize, impacting their families and neighbours who depended on them. Also, during the drought, vegetable production collapsed as 10% of the participant reported to have been supplied less than 25% of their household needs. Essentially, the 2015-16 droughts gravely impacted water supplies in the study area, affecting irrigated gardens to the extent that they also failed thus compromising food security for farmers and their neighbours
22	Quinn, C. H., et al., 2011.[68]	Mixed-methods approach using purposeful sampling for individual interviews, preference survey and Focus Group Discussions	n= 17 interview participants (14 women and 5 men); FGDs (n=100); Preference survey (n=650); and semi-structured interview (n=9) with government officials from the Sekhukhune district	South Africa: Sekhukhune District, South Africa.	Investigate how local communities cope with and adapt to multiple stresses in rural semiarid South Africa.	Drought, maize prices, ill-health	Drought was perceived to impact households by influencing higher maize prices in local markets, reducing home-gardens food production, and increased disease prevalence. Coping strategies included: adjusting food intake levels, finding work to reduce stress, spending less on food or eating less, social networks (borrowing food or sending family members to eat with those better-off), and rural urban migration). Approaching the district for help, either directly or through support for community projects, or accessing government grants were both identified as strategies for dealing with difficult circumstances.
23	Schmidt, M. and O. Pearson, 2016.[69]	Qualitative study design using interviews with various stakeholders to assess challenges to local livelihoods in the Afar region of Ethiopia.	Ethnographic rural fieldwork included 20 semi-structured Qualitative group interviews (12 male, four female, and four male and	Ethiopia (Western Afar region in four kebeles (villages) of Ewa and Awra	Investigates the impact of environmental, institutional and cultural changes on natural resource management strategies, using empirical	Drought, Erosion,	Drought, erosion and associated impact on rangelands and pasture for livestock impacts negatively on the population of herds in the region as well as natural resource management. Stakeholder-

			female) and 27 in-depth, semi-structured Qualitative interviews with key informants (22 male, five female). 15 IDIs (14 male, one female) and one FGD	Woreda (district) within Zone Four.	research undertaken in four villages of western Afar (Ethiopia) to assess the related challenges to local livelihoods		interviews reveal that the authority and use of traditional common property regimes have been considerably diminished and traditional livelihood practices threatened as a result of on-going land privatisation and an increased government presence in the region.
24	Brown et al 2011.[73]	Quantitative (longitudinal) study: National level precipitation statistic and spatial and temporal variability for Sub-Saharan Africa.	n=42 sub-Saharan African countries (1975 – 2003).	NIL	Examines evidence of the effects of climate change on economic growth in Sub-Saharan Africa (SSA).	Drought, Temperature and Precipitation	Drought has the most significant climate influence on GDP per capita growth, while Temperature and precipitation variability show significant effects in some cases. Increases in temperature have a significant and substantial negative impact on agriculture as a one-degree celsius increase in average annual temperature is associated with a decrease in the GDP from agriculture of approximately 11%.
25	Dile et al 2016.[74]	Quantitative (Geo-spatial) study using the Soil and Water Analysis Tool (SWAT) including a digital elevation model (DEM), stream network, soil, and land cover. Weather data from the Ethiopian National Meteorological Services Agency.	The weather data consisted of daily rainfall and maximum and minimum temperature.	Ethiopia: Megech watershed, North Gondor administrative zone.	Develop a decision support system in a meso-scale watershed within Lake Tana basin to help determine suitable areas for locating ex-situ water harvesting systems and the corresponding sizes of the water harvesting ponds	water harvesting	Supplementary irrigation in combination with nutrient application increased simulated *Teff* (a staple crop in Ethiopia) production up to three times, compared to the current practice. Moreover, after supplemental irrigation of teff, the excess water was used for dry season onion production of 7·66 t/ha (median). Results suggest that water harvesting can be important in increasing local-to regional-scale food security through increased and more stable food production and generation of extra income from the sale of cash crops.
26	Gao & Mills 2018.[75]	Quantitative study; Strategic random sampling of households in over 15 years span. Panel Data was merged with village level climate data.	Sampled population for study analysis (n=5038) was drawn from selected households in the 15 rural Ethiopian villages.	Rural Ethiopia	Estimate: the impact of weather shocks on consumption and poverty dynamics in rural Ethiopia; and the effectiveness of household coping strategies in alleviating the impacts of shocks.	weather shocks: drought, increased rainfall; safety nets	Increased temperature (high temperature linked to drought) negatively impacts consumption as opposed to increased rainfall which seems to bud well for adult consumption patterns. Where there are formal social safety net transfers, rainfall shocks impact on consumption are mitigated; and off-farm employment is used during high temperature shocks as well.
27	Twongyirwe, R., et al., 2019.[76]	Quantitative Exploratory study using survey data from 140 farmers.	Of the 140 respondents, 57.9% (n=81) are female while 42·1% (n=59) are male.	Uganda: Isingiro District	Characterise relationships between perceptions of drought and food insecurity and household coping responses; compare livelihood features of	Drought	Among the subset of households that perceived food insecurity to be a problem (n=96), 46% and 54% explained food insecurity as the result of

					farmers that perceived food insecurity as a problem and those who did not; and investigate how household-level characteristics correspond to household coping strategies.		drought that caused either total crop failure or reduced yields, respectively.
28	Cafer, 2018.[77]	Mixed methods: Surveys with farmers; and interviews. Purposive sampling was used for the survey because of tensions between farmers and extension services.	Study population (n=115 farmers) in the Amhara region of Ethiopia.	Ethiopia: Amhara region (South Wollo zone).	Explores khat production as an adaptive strategy situated within a larger resilience strategy. Specifically, khat's potential for improving short-term food and economic security, juxtaposed against its potential for increasing vulnerability through reduced health outcomes and natural resource degradation.	khat production, drought	*Khat* was positively associated with food security. It was a resilience (adaptation) strategy against climatic and economic shocks since it provided farmers (and households) with income to cushion various shocks. However, because *Khat* is water intensive, there is reason for caution given the documented potential for poor health outcomes related to khat consumption and the heavy reliance on irrigation for intensive khat production, combined with the unregulated nature of water withdrawals in Ethiopia.
29	Nnadi, O. I., et al., 2019.[78]	Mixed methods: Meteorological data Household survey, climate data; Field observations, Household questionnaire, FGDs and KIIs.	Men and women household heads (150 each) were purposively used in order to get detailed information in the households. Separate FGD session of eight members each (including leaders of farmers' cooperative groups) were organized for men and women farm household heads in each Local government area (LGA). Key informants of men and women included four (n=4) community leaders with in-depth knowledge of the area.	Nigeria: (Three LGAs (Anambra East, Ogbaru and Ayamelum) in Anambra state.	Examines: perceptions and experience of men and women farmers on the impacts of Climate Variability and Change (CVC), using climate data and farmers' perception from a gender perspective; and the effect of CVC on annual and seasonal rainfall, number of rain days, onset and cessation of rainfall; perceived impacts of CVC on annual and seasonal rainfall and compared the measured (meteorological) impacts with the perceived impacts across gender.	Climate Variability and Change (CVC) and Gender	More dry, than wet, years occurred in the three locations (Ogbaru, Anambra East and Ayamelum); Seasonal rainfall was unevenly distributed from 2007-2016; and Men and women responses revealed that there were statistically significant changes in the onset of rainy season, early cessation of annual rainfall; alteration of growing seasons; frequent flooding and frequent drought. Women felt more impact of food insecurity, water shortage and had more burden of migration due to changes in rainfall. Gender was central to the experiences linked to the study.
30	Speranza, C. I., et al., 2008.[79]	Mixed methods: Data from a longitudinal survey of 127 households, interviews, workshops, and daily rainfall records (1961-2003) were analysed using Quantitative and Qualitative methods	Participants consisted of n=127 households from 8 villages	Makueni District, Kenya	Examines whether, and how crop production conditions and agro-pastoral strategies predispose smallholder households to drought-triggered food insecurity.	Drought	During the 1999/2000 droughts, 91% of the households experienced food shortages, on average of 3 months in 1999 and 5 months in 2000. Some households could not produce enough food for own consumption, and as a result they had to purchase food to supplement own production. Some sold their produce at low prices immediately

							after harvest and purchase them later at higher prices.
31	Gwatirisa and Manderson, 2012.[80]	Qualitative design; snowball sampling was used to identify households; ethnographic observation was conducted as well as Key Informant Interviews as part of the data collection techniques	Twenty-five households were recruited into the study; the inclusion criteria were that the primary caregiver to a PLWHA was from a household where the person designated head of household was salaried, and, therefore, the household was ineligible for food aid; this was the case even when the head of household was the care recipient.	Zimbabwe Mutare, southeast (urban) Zimbabwe	To show how serial drought, poor crops and a web of national and global historical, economic, political and social factors imploded to produce unprecedented crisis that affected the lives of residents of Mutare, particularly households with specific urgent food needs for people with AIDS.	Drought, food insecurity, HIV	Drought is linked to crop failure and poor economic status, nationally and at household level; impacted negatively on individuals, families and especially people who care for HIV infected relatives. Some of the coping mechanisms adopted included crossing the border with Mozambique to procure food (and other) commodities that were lacking in the Mutare area.
32	Davies S 2010.[82]	Quantitative study: Secondary panel survey data.	n=2355 (rural) households between 2000 and 2002 who experienced whether shocks and lacked adequate financial infrastructure.	Rural Malawi	Explore long-run impacts of two household shocks (sickness and death) and two community shocks (floods and drought) on household per capita consumption.	Drought, health status, flooding	In rural Malawi, sickness and droughts have a short-run negative impact on per capita consumption levels, but no persistent impact. Flooding has no immediate impact, but a positive longer-run impact, as many households in the flooding zone actually benefit from increased rainfall.
33	Baudoin et al., 2017.[83]	Mixed methods (multiple data sources): literature reviews from 1920 in drought responses; key informant interviews and surveys with various key actors managing drought policy.	Interview participants (n=10) included policy makers, climate scientists, representatives of the private sector, civil society and government departments.	South Africa	Analyse drought management in South Africa with a focus on the El-Niño-related drought 2015-16; investigate the robustness of the institutional and policy responses to address severe droughts in the country; and identify possible lessons emerging from the drought to enhance preparedness for future climate-related stresses.	Government's drought response mechanisms, Drought	Drought response in South Africa are negatively affected by factors such as bureaucratic processes as well as lack of preparedness. In fact, bureaucratic inability to enable swift and flexible responses resulted in many NGOs and civic actors stepping up to provide assistance.
34	Silva, J. A. and C. J. Matyas, 2014.[85]	Quantitative study using longitudinal data on rural households to assess impacts of rainfall patterns on agro-income	Rainfall zones: 1 (n = 205); 2 (n= 425); 3 (n = 455); 4 (n =711); 5 (n= 882); 6 (n=196); 7 (n=347); 8 (n=355); 9 (n=283)	Mozambique: Nine Rainfall zones	Examine the effects of distinctive rainfall patterns on (agricultural) crop income while controlling for agricultural and demographic characteristics of households.	Rainfall variability	Results show that in a period where monthly rainfall seldom occurred in normal amounts, most households experienced decreases in agricultural income. Even after controlling for rainfall patterns, they find that greater household dependency on staple crop

							agriculture is associated with declining annual agricultural income
35	Hlahla & Hill, 2018.[86]	Mixed methods: Structured questionnaires and IDIs.	Surveyed (n=378 households) - 61% were female-headed and respondents. 70% of respondents were female and the majority of respondents were within the 16 to 45 and over 65 age groups.	South Africa: KwaZulu Natal Province (Pietermaritzburg)	Investigate the impacts of climate variability on marginalized urban communities within the city of Pietermaritzburg, and to investigate how these communities are responding.	Climate variability	60 percent of participants identified drought as a major climate stressor that negatively affected their agricultural activities (maize, cabbage, spinach, potatoes, beans, butternuts, beetroot, carrots and onions); 24% report (negative) health impacts; and Rural Food insecurity resulting from drought causes urban population to send remittances to rural communities to purchase food.
36	Speranza, C. I., 2010.[87]	Mixed methods: Data were collected from a longitudinal survey of 127 agro-pastoral households. FGDs and Expert interviews were also conducted	Participants consisted of n=127 households from seven villages.	Kenya (Makueni district).	Analyses how agro-pastoralists in Makueni district Kenya, adapt their livestock production to climate variability and change.	climate variability and climate change; poverty and lack of adaptive capacity	Approximately one-third of the households have inadequate feeds, and livestock diseases are major challenges during non-drought and drought periods. Agro-pastoralists' responses to drought mainly involved intensifying exploitation of resources and the commons. Poverty, limited responses to market dynamics and inadequate skills constrain adaptations.
37	Mussa, F. E. F., et al. 2015.[92]	Quantitative: Geo-spatial and modelling techniques	Unclear	South Africa (Crocodile River catchment)	Assess drought intensity and severity and groundwater potential for use supplementary source of water to mitigate drought impacts in the Crocodile River catchment in South Africa.	Drought	Low-rainfall areas showed more vulnerability to severe meteorological droughts. The most water stressed sub-catchments with high level of water usage but limited storage, such as the Kaap located in the middle catchment and the Lower Crocodile sub-catchments, are more vulnerable to severe hydrological droughts.
38	Ferrer et al 2019.[93]	Quantitative (Environmental) study using geological data collection techniques.	In order to estimate the effect of La Niña drought on the seasonal and annual recharge patterns, groundwater recharge was estimated for the period 2012 to 2017 from the daily soil water budget.	Rural area: Kwale County	Define the hydrodynamics of the Kwale hydrogeological system; and show the effects of the La Niña 2016/17 drought on the groundwater system.	Weather shocks: drought and flood	Drought triggered a 69% reduction of groundwater recharge compared to an average climatic year. There was reduced recharge during the first rainy season (April–June) and no recharge during the second wet season (October–December).

39	Hove et al., 2019.[94]	Qualitative (case) study: Participatory Action Research (PAR) based on workshops, Photovoice and thematic analysis.	25 participants were drawn from 3 villages	South Africa: Rural Province of Mpumalanga	To develop local knowledge on health priorities in a rural province as part of a programme developing community evidence for policy and planning.	Drought	Drought was identified as one of the serious exacerbating factors linked to water insecurity in rural South Africa. Low water quantity and quality - empty reservoirs and dried rivers - as well as problems of poor service delivery, poor infrastructure and health challenges are heightened by Drought.
40	Bola et al., 2014.[95]	Mixed methods: Semi-structured interviews, FGDs, and surveys focused on rainfall variability and peoples' coping strategies	Study population (n=144 households)	Zimbabwe (Mbire district) embedded in Southern Africa analysis.	Examine whether and in what ways extreme weather events impact on (people's) livelihoods	Drought and Floods	Individual and Household Coping strategies during drought and floods included vegetable farming and crop production in the floodplain, taking on local jobs that brought in wages, planting late' livestock disposals and daily out-migration to Zambia or Mozambique.
41	Markantonis et al., 2018.[96]	Mixed methods: combined geo-spatial gridded climate data and Quantitative and Qualitative household surveys focused on three countries sharing the Mékrou River basin.	Study population (n=660) consists of households randomly surveyed from villages in the three countries.	Benin, Burkina Faso, and Niger	Assess the occurrence of flood and drought events, estimate damage costs at the household level, and describe the current mitigation behaviours adopted by the population of the Mékrou River basin, a small catchment area in West Africa.	precipitation, temperature, and river discharge	Burkina Faso experience was mainly agricultural production losses whereas for Benin, the drought impacts manifested as agricultural production, livestock losses and malnutrition.
42	Gray & Mueller 2012.[112]	Quantitative design: Panel data drawn from the Ethiopian Rural Household Survey collected over a period of 15 years from 15 villages.	Study sample draws from n=1500 households over the ten-year period.	Ethiopia: Ethiopian Highlands	Investigate the effects of drought on population mobility over a ten-year period in the Ethiopian highlands	Drought	In times of drought, men migrating out searching for work (labour-migration) increases. Also, household lacking land are mostly most vulnerable to drought.

43	Henry et al 2004.[113]	Quantitative study: Combined longitudinal multilevel; national retrospective migration survey; and rainfall time-series data.	Analyses are restricted to the first out-migration from the village after age 15, retained as the age at which participation in decision-making is considered to commence	Burkina Faso	Investigate the impact of rainfall conditions on the risk of the first village departure in Burkina Faso	Rainfall variability, community and individual level characteristics	Migration as the effect rainfall variability (or drought) in Burkina Faso is only significant when it is about moving from one rural area to another (but not internationally). People in drier regions in this case are more likely than those from wetter regions to migrate temporarily or permanently (to other rural areas). But independently, there is not a direct effect of drought on migration.
44	Nawrotzki, R. J. and M. Bakhtsiyarava, 2017.[114]	Quantitative study using representative census data combined with high-resolution climate data derived from the novel Terra Populus system.	n = 133 686 household in Burkina Faso; and n= 57 052 household in Senegal	Burkina Faso (45 provinces); and Senegal (31 departments)	Explore climate-migration relationship in rural Burkina Faso and Senegal.	Temperature and precipitation extremes	Excessive precipitation increases international migration from Senegal while heatwaves decrease international mobility in Burkina Faso, providing evidence for the climate inhibitor mechanism. There is a conditional effect of droughts on international out-migration from Senegal, which becomes stronger in areas with high levels of groundnut production.
45	Owain, E. L. and M. A. Maslin, 2018.[115]	Quantitative study: Composite conflict data of political violence (MEPV) compared with climatic, economic and political indicators using optimisation regression modelling.	Data is drawn from 10 East African countries spanning from 1963-2014	East Africa	Analyse whether climatic changes between 1963 and 2014 impacted the risk of conflict and displacement of people in East Africa	Population growth, economic growth and the relative stability of the political regimes.	Climate variations did not significantly impact the level of regional conflict or the number of total displaced people (TDP); variations in refugee numbers were significantly related to climatic variations as well as political stability, population and economic growth. Also, long-term population growth, short-term negative economic growth and extreme political instability seem to be primarily linked to conflict. This study suggests that climate variations played little or no part in the causation of conflict and displacement of people in East Africa over the last 50 years.
46	Simatele, D. and M. Simatele, 2015.[116]	Qualitative (Participatory research methodological) design	Participants consist of n=30 Tonga Migrant households that moved from the Southern Province to the Central Province	Zambia (Mukonchi and Lunchu settlements in Central Province of Zambia	Investigate factors and processes that motivated and triggered the relocation of the 30 migrant households from four districts in Southern Province to Lunchu and Mukonchi in Central Province	Effects of extreme weather conditions (drought, flooding and extreme temperature)	Participants' interview revealed that droughts not only compromised their livelihoods but also eroded some of their productive assets. Coupled with poor rainfall, drought compromised the individual and community capacity to obtain meaningful livelihoods as agricultural productivity, livestock survival and household assets that facilitate resilience against climate change were strained. To cope, people opted to relocate to central Zambia

							looking for better livelihood options. Migration thus served farming households' quest to mitigate climate change impacts.
47	Little PD, 2008.[117]	Mixed methods: Simple random sampling of households; case studies of selected households; detailed interviews	416 households randomly selected (n=416); selected case study households (n=62).	Ethiopia: The South Wollo region.	Investigates if food aid due to drought (in South Wollo, Ethiopia) leads to dependency both in terms of its effects on local economic and social behaviour; and its contribution to aggregate food supply at community and household levels	Drought and Food aid	Food aid recipients did not change their social and behavioural strategies of coping/adapting to the drought (shocks) in contrast to views that food aid encourages dependency. Data showed that despite interventions, more recipient households continued to engage in off-farm waged employment and other petty business activities. Piecemeal jobs such as labourer, street hawking and gathering and selling firewood and charcoal remained pronounced among members of these families as part of mitigating the impacts of drought on their income and food security. These activities are particularly pronounced during droughts when food aid availability is higher than in other periods.
48	Linke et al 2018.[118]	Quantitative study: Combined national survey (2014) and spatial data on rainfall trends. Simple random sampling was used for identifying households.	Study population (n= 1400) participants of the national survey in 2014	Kenya	Understand conditional social contexts that shape attitudes toward violence under drought conditions.	Drought	Statistical models show some evidence that drought modestly increases the level of support for violence among Kenyans. Some models also indicate that areas with drought are more likely to experience violent events during the year after survey data were collected.

49	Jones et al 2017.[121]	Quantitative (cross-national) study of violent uprisings, food security and vulnerability etc.	Unclear	Africa-wide	Exploring the effect of food insecurity and state vulnerability on the occurrence of violent uprisings in Africa	Government's capacity and susceptibility to food insecurity	Violent unrest is the product of both food insecurity and the underlying vulnerability of the state to shocks, but vulnerability impacts unrest more than food insecurity.
50	Bosongo et al., 2014.[122]	Mixed methods: Semi-structured interviews, KIIs and FGDs. Sampling was purposeful.	Study population: Survey (n=144 participants) 90 male and 54 female; KIIs (n=5); and 2 FGDs.	Congo, Democratic Republic.	Analyse how floods and droughts affect communities' livelihood in the middle Zambezi river basin and coping mechanisms which households apply to counter the impact of floods and droughts.	Drought and Floods	The impacts of floods and droughts in the district, notably in some wards such as Kanyemba, are the reduction of crop production, food shortages, reduction of agriculture-derived income and erosion of social network. Households coping mechanisms included assets disposal, labour migration, stream bank and floodplain cultivation, piecework, remittance, wild production and fishing.
51	Antwi-Agyei P et al., 2014.[123]	Mixed methods: Surveys, key informant interviews and participatory methods.	Unclear	Ghana: The Sudan savannah and forest-savannah transitional agro-ecological zones	Examine adaptation/coping strategies by farming households in Ghana to mitigate the adverse effects of climate variability on their livelihood activities.	Rainfall variability, Drought	On-and-off farm adaptation strategies found in the study include changing the timing of planting, planting early maturing varieties, diversification of crops, support from family and friends, and changing diets to manage climate variability. Most households decided to engage in multiple non-arable farming livelihood activities in an attempt to avoid destitution because of crop failure linked to climate variability (particularly drought).
52	Unks, R. R., et al., 2019.[124]	Mixed methods: Survey, IDI and FGDs data were used together.	Survey data (n=214) excluded people without livestock and incomplete survey response. FGDs (n=8) with men and women; and IDIs (n=21) with senior elders about herding ecology and livelihood changes over the previous 30 years.	Central Kenya	Observe how changes in formal and informal institutions have differential impacts across populations in terms of vulnerability of livelihoods to drought, and the unequal processes that shape adaptation to new conditions	Exposure to drought	Herders with higher livestock wealth had access to secure cattle grazing on private wildlife conservation lands, and to more distant areas with herds of sheep and cattle – two key means of reducing exposure to drought vulnerability – leading to greater drought coping capacity. Those with lower livestock wealth rely disproportionately on illicit, precarious access to external grazing resources. Higher livestock wealth families experienced disproportionately lower sensitivity to drought with smaller losses of cattle, and likely have decreased sensitivity to drought-related market fluctuations, while others are

							primarily reliant on small stock and/or precarious access pathways.
53	Nalley, L., et al. 2018.[137]	Quantitative study using farmers' annual crop yield data	n= 36 507 crop yield from n=125 test plots across South Africa (1998-2014) used to model yield gains. The dataset includes n=26 crop varieties (16 spring, 5 facultative, and 5 winter) commercially released cultivars.	South Africa	Determines proportions of observed yield increases in released spring, facultative, and winter wheat cultivars that are attributable to genetic improvements by the South African Agricultural Research Council's Small Grain Institute (ARC/SGI).	Drought-related response (adopting the Agricultural Research Council (ARC) Genetically Modified Wheat cultivars); and it's on farmers' yield	South African farmers who adopted the ARC's wheat varieties experienced an annual yield gain of 0·75%, 0·30%, and 0·093% in winter, facultative, and irrigated spring wheat types, respectively.
54	Fisher et al 2015.[138]	Quantitative design. Data from household surveys conducted initially in 13 Sub-Saharan African countries, with the study focused on 6 eastern and southern African countries.	Study population (n=3700) draws from farm households ranging from 400-600 in the selected countries	Ethiopia, Tanzania, Uganda, Malawi, Zimbabwe and Zambia	Identify future investments that will stimulate Drought Tolerant Maize (DTM) adoption, and increase farmer resilience to drought, in sub-Saharan Africa	Age, sex, and education attainment; household members working; agricultural resources (total cultivated area); access to credit to purchase maize inputs; access to information about new seeds	Uptake of the DTM in selected countries varied but overall uptake was hampered by: Unavailability of improved seed; Inadequate information (sharing); lack of resources (poverty); high seed price; perceived attributes of different varieties of the DTM.
55	Makate, C., et al. 2019.[139]	Quantitative study. Simple random sampling used to select districts in selected provinces in Zimbabwe and Malawi.	Study population (n=1172): smallholder farmer households from Malawi (n= 572) and Zimbabwe (n=600).	Malawi and Zimbabwe (4 districts each)	Evaluate factors that explain individual and multiple adoption of climate change management strategies and their differential impacts on productivity and income	Conservation Agriculture, Drought-Tolerant Maize, Age, Land size, access to credit, access to extension services, access to information, education level	Access to credit, income and information; education levels and the size of individual or smallholder-owned land affected their adopting innovative conservation agriculture. However, among the adaptation strategies - ranging from conservation agriculture to the use of stress-adapted legume varieties and drought-resistant maize species - the most effective adaption strategy with a higher chance of improving productivity involves the simultaneous application of all the adaptation strategies together.
56	Nonvide, G. M. A., et al., 2018.[144]	Quantitative study: Structured survey, and Stratified random sampling used to select the survey respondents	n=690 participants (rice producers); n=540 dry-land farmers; and n=190 irrigators	Benin (Four districts (Garou, Guene, Malanville and Tombouctou) in	Analyze farmers' perceptions about the use of irrigation for rice cultivation and the constraints faced by farmers in	Specific constraints in the irrigation scheme of Malanville include the high cost of	Farmers' positive perceptions of irrigation include the use of irrigation for insurance against drought, crop yield improvement, higher income, food

			drawn from the 4 districts that participated in the survey. were selected.	Malanville municipality, Benin.	producing it. Specifically, to understand farmers' perceptions about the use of irrigation; identify the constraints faced by farmers in rice production; propose suitable policy responses to overcome those constraints.	irrigation and unavailability of water.	security and poverty reduction. Constraints include expensive irrigation; water unavailability; lack of agricultural credit, poor access to production inputs, inadequate knowledge of water resources management, poor access to agricultural information and markets, and flooding of fields.
57	Asare-Kyei D et al., 2017.[140]	Mixed methods: Combined participatory approaches with statistical, remote sensing and GIS techniques to develop community level vulnerability indices in three watersheds (Dano, Burkina Faso; Dassari, Benin; Vea, Ghana).	Study Population (n=432) disaggregated by watersheds: Vea (n=240 households); Dano (n=100) and Dassari (n=92 households).	West Africa (West African watersheds in Burkina Faso, Benin and Ghana).	Quantify risk and vulnerability of rural communities to drought and floods.	Extreme climatic (drought and flood) conditions	High risk areas experience factors such as: high exposure to droughts and rainstorms, longer dry season duration, low caloric intake per capita, and poor local institutions.
58	Adgo E et al., 2013.[141]	Mixed methods: Data drawn from government records, observation and questionnaires administered to farmers.	About 60 farmers were interviewed. Half of the respondent had terraced farms in the watershed former project area (with technology) and the rest were outside the terraced area (without technology). Crops assessed: Teff, Barley and Maize.	Ethiopia: Anjenie Watershed.	Determine the economic benefits with and without terraces, including gross and net profit values, returns on labour, water productivity and impacts on poverty.	Soil conservation through the terracing of farming areas	The soil conservation strategy employed in the pilot areas had significant positive effects on the productivity of crops and the overall income of families and food security. It also reduced soil erosion.
59	Fisher & Carr, 2015.[142]	Mixed methods: Survey data; KII and risk elicitation experiment.	Study population (n=408 households) and (n=696 individuals.	Uganda: Eastern Uganda.	Examine how gendered roles and responsibilities influence adoption of drought-tolerant (DT) maize, a new technology that can help smallholder farmers in sub-Saharan Africa adapt to drought risk.	Gender roles	Compared to men, women farmers have much lower adoption of the DT maize mainly because of differences in resource access, notably Land, Agricultural information and Credit (facilities).
60	Mavhura, E., et al., 2015.[143]	Mixed methods: Meteorological data linked to Qualitative and Quantitative interviews.	Study population (n=60) was drawn from household heads in the communities within the study area.	Zimbabwe (Zambezi Valley, Mashonaland Central Province).	Investigate drought impact on food security and smallholder farmers' coping strategies in the Zambezi Valley	Drought	Participants' identified drought-related impacts entailed: increased food shortages; reduced household consumption levels, including diet changes; increases in food prices, and fall in livestock value; sale of livestock to mitigate drought impact; trade-offs between savings and meeting households' food consumption needs. Essentially, drought stifled food production within communities in the Zambezi Valley, especially during the 2011/2012 season by affecting their agricultural production system. Community coping strategies included:

							adoption of drought-tolerant crop production; crop variety diversification; purchasing cereal through asset sales; NGOs food aid and gathering wild fruits.
	**Drought-Health**						
61	Anthonj, C., et al., 2019.[4]	Mixed methods: Cross-sectional surveys; IDIs and KIIs.	Survey population: n=400 household heads; smallholder (n=106) and commercial farmers (n=95), nomadic pastoralists (n=99) and service sector workers (n=100) in the Ewaso Narok Swamp. IDIs consisted of (n=20 in total); expert KIIs (n=8).	Kenya: Ewaso Narok Swamp, Laikipia county.	Assess the risk perceptions towards infectious disease exposure in the Kenyan Ewaso Narok Swamp and evaluate whether the perceived risks reflect the actual risk factors	Wetlands and diseases; drought (dry season) and socioeconomic and mental health impact (stress associated with the loss of cattle - source of livelihood - due to lack of water)	Among other things, including health risks associated with living in wetlands, drought was also identified, mostly among the pastoralists, to affect people in many ways including socioeconomic and mental health impacts due to the loss of their main source of livelihood - their cattle. Losing livestock drought was devastating for pastoralists in general.
62	Burke, M., et al., 2015.[8]	Quantitative (Cross-sectional) secondary data analysis from 21 Demographic Health Surveys (DHS) of 19 Sub-Saharan African countries	Study population: Individuals (n=2000) across 19 African countries used to compare the HIV status of individuals randomly exposed to a higher number of recent shocks (past 10 years) to the status of nearby individuals exposed to fewer recent shocks.	Africa-wide (Rural and Urban)	Assess the role (and or effect) of negative income shocks on HIV outcomes across the African continent.	Drought, rainfall shocks, Gender	Exposure to recent negative rainfall shocks substantially increases HIV infection rates in rural areas with high baseline HIV prevalence. Exposure to a single additional shock leads to a significant 11% increase in overall HIV infection. However, shocks do not appear to induce earlier marriage or increased time away from one's village. But, the effects of shocks on HIV are larger for men working outside of agriculture (whose purchasing power would have declined the least), evidence that is broadly consistent with an outward shift in the supply of transactional sex.
63	Brown, A. L., et al., 2016.[70]	Quantitative (Experimental) study	Cassava under different conditions of drought and temperature versus better watered categories	NA	Determine the separate and interactive effects of temperature and drought on the growth and toxicity of cassava.	Temperature and drought	The study found that tuber yields were increased and CNp (Cynaide potential) was decreased when plants were grown under higher temperatures with adequate water supply; however, the combined effects of higher temperature with drought stress had a negative effect on tuber yields and nutritive value.
64	Nawrotzki, R. J., et al., 2016.[71]	Quantitative study: Combined socio-demographic and climate change data for climate-migration nexus. Secondary data (TerraPop, 2006 and 2002)	Analytical sample (n=164 884 households) in Burkina Faso and (n=57 052 households) in Senegal. The climate information available via TerraPop	Burkina Faso and Senegal	Investigate whether: climate effects on international migration differ by the local food-security context; climate variability is more strongly associated with international migration in highly food	drought, precipitation, food insecurity	Generally, the local food security context was significantly linked to migration probabilities. Households residing high versus low levels of (growth) stunting are likely to send a member to an international destination. In Burkina Faso, international

			covered the years 1900 to 2013.		insecure regions of two west-African countries: Burkina Faso and Senegal.		outmigration fell as heatwaves increased since its impact on production and resources limits households' capacity to send someone abroad. Conversely in Senegal, increased precipitation was associated with more international outmigration as it coincided with boom in production and income for households, thus increasing their capacity to send someone abroad.
65	Hennink, M. and McFarland, D. A., 2013.[72]	Qualitative (Grounded theory-exploratory) study: IDIs, FDGs among recipients of microcredit.	Study participants were recipients of microcredit from a government microfinance institute, FAARF. IDIs (n=18); and FDGs (n=10) with 6-8 participant in each group.	Burkina Faso: Ouest (west) region.	Explores how microcredit enables changes in health behaviour.	Microcredit, economic shocks	Although microcredit facilities usefully facilitated savings, investments in household health, including initiating health prevention and readiness for certain emergencies, economic shocks such as those from drought negatively affected their resilience - eroding some, if not all the, gains made in health. Periods of drought that lead to crop failure affected women whose business activities relied on agriculture, as well as those who bought and sold agricultural produce for profit. Qualitative data revealed that during these times little or no profit was made, and women often had to secure a supplementary loan to make repayments on their initial microcredit loan, placing additional financial hardship on households and making health expenditure unfeasible.
66	Lawson, D. and Kasirye, I., 2013.[81]	Mixed methods: Longitudinal panel data with IDIs to examine household coping strategies.	146 households were interviewed in both 2005 and 2009, all of whom were part of the original 1992–1999 household panel survey for Uganda.	Uganda	Examine how households cope with financial shocks (such as losing assets and jobs) and analyse how these compare with the dominant non-financial shocks such as illness and death prevalent in many SSA countries as a result of the HIV/AIDS pandemic.	Ill-health, Economic, agricultural and domestic shocks	Larger households are significantly more likely to engage in asset sales than reduce consumption. Similarly, households with very many dependants, aged below 18 & above 60years, are less likely to reduce consumption when faced with shocks. Also, shocks such as Drought makes the reduction of consumption more likely while Floods more likely leads to the sale of assets as part of households coping strategies. Ill-health experienced over the 1992–1999–2005–2009 period leads to a reduction of assets over time.
67	Coppock, D. L. and Desta, S., 2013.[88]	Mixed methods: Surveys; interviews and purposeful sampling of women in 16	Study population (n=344) women who belonged to various groups.	Kenya: Marsabit District.	Understand how and collective action groups (in North-central Kenya - a pastoralist region	Drought, poverty, resource scarcity,	Collective action groups emerged with encouragements from Government Development agencies or NGOs;

		settlements deemed feasible for the study.			characterised by high poverty rates, droughts, ethnic conflicts etc-) were formed and how they function(ed) to mitigate and adapt to stressors such as drought.	unfavourable group dynamics, illiteracy	Founders were predominantly women, mostly illiterate, poor, married and from similar ethnic groups; Group action was not very active at initial drought (1990s) though some stock redistribution to poor households and low interest loans granted to members; More formal steps were taken including anticipating droughts, selling excess stocks in advance when prices are higher and investing proceeds in small businesses or improving living standards in community; and restocking through business profits when rainfall conditions improve.
68	Bahta, Y. T., et al., 2016.[89]	Quantitative (case) study: Semi structured Questionnaire and Rapid Rural Appraisal (RRA) used for data collection and deeper contextual grasp of drought perceptions.	The communal farmers sampled (87) were those who were willing to participate in the survey, after the purpose of the study was explained to them in a workshop.	South Africa: O.R. Tambo District, Eastern Cape province.	Assess the farmers' perception of agricultural drought, with an insight into drought vulnerability to their farming operations, gender, social network, role of government, stress and security and safety.	Drought, gender, social networks, the role of government, stress, and security and safety related to drought.	The results revealed that perceptions held by communal farmers indicate that (i) they receive inadequate government support for drought risk reduction, (ii) they do not consider social networks as being effectively involved in drought risk reduction, (iii) there is a system of gender stereotyping among the farmers with discrimination against women, (iv) psychological stress affects their farm activities, and (v) they experience high levels of stock theft and insecurity in their farming.
69	Abiona, 2017.[99]	Quantitative (Cross-sectional Cohort) study: Household survey data Pooled for the years 2004-5, 2011 and 2013.	Sample population: n= 11,280 households and n=49 066 individuals (2004-5); n=3246 households and n=15 582 individuals (2010-11); and n=4000 households and n=20076 individuals (2013).	Malawi (Rural settings)	Investigate: the impact of weather variation and extreme weather events around the time of birth on adult welfare outcomes; and the persistent impact of early life harvest driven rainfall (drought and flood-related) shocks on childhood and adulthood welfare outcomes such as Health, schooling and (human) satisfaction levels.	Rainfall (flood) and drought shocks,	Adults who experienced drought and rainfall shocks early in life were more likely to have greater school entry delays and be unhappy with their current economic situations. The effects of drought shocks were found to be more persistent than flood shock incidence in early life.
70	Dinkelman, T., 2017.[101]	Quantitative (Longitudinal Cohort) study. Secondary (census data) and analysis based on 10% individual record data from the 1996 South African Census	Study population (n=655 532) disaggregated by Gender (males n=298 475, females n=357 057)	South Africa: Previous apartheid homelands.	Estimate the effects of Early-childhood drought exposure on later-life disabilities among South Africans confined to the homelands during apartheid.	Drought, gender	Drought exposure raises the prevalence of all types of disabilities, and more impacts are experience by males than females.

71	Linke, A. M., et al., 2015.[119]	Quantitative (Cross-sectional) study using survey data and multilevel analysis	Study population (n=504 individuals) surveyed for the study.	Kenya: Nakuru, Uasin Gishu, and Vihiga counties in the Rift Valley and Western areas of Kenya.	Clarify some of the linkages between environmental variability, especially precipitation, on violent conflicts with a consideration toward the possibility that intervening and moderating societal influences might affect such a linkage.	Drought frequency and severity, informal dispute management institutions; and politics	Frequent droughts (over 10 years) lack statistical support to exacerbate conflict. Formal and institutionalised governmental rules don't moderate or reduce the potential for drought to elicit increased support for violence. Instead, where drought is reported to be getting worse, there is evidence that inter-community dialogue is associated with lower levels of support for the use of violence. Essentially therefore the value of community dialogue cannot be under-estimated in the promotion of peace during resource scarcity.
72	Eissler, S., et al., 2019.[120]	Quantitative (Cross-sectional) study from 40 rounds of DHS data (1990-2015) linked to high resolution precipitation and temperature records to explore climate-fertility choice nexus in sub-Saharan African (SSA).	Study population (n=70 879): reproductive age women (15-49 years) who are married or cohabiting for the first time with 10 years before the survey.	18 SSA countries.	Examine the overall association between temperature and precipitation anomalies and both fertility ideals and preferences among sub-Saharan African women.	Temperature and climate variability - drought, floods.	Women who are exposed to above-average temperatures report lower ideal family size and reduced probability of desiring a first and additional child. Precipitation anomalies (drought or flood) during 12 months prior to the DHS survey was associated with a significant reduction in ideal family size But longer spells (about 60-months) of above average precipitation was associated with increases in the ideal family size.<br>Generally, women were therefore found to significantly prefer to adjust their fertility downwards during times of unfavourable environmental conditions, particularly hot spells, while responses to precipitation vary over the short and longer run.
**ART-Adherence**							
73	Haberer J et al., 2019.[32]	Quantitative	Study sample size (n=904)	Uganda and South Africa	Explore ART adherence among individuals with early-stage HIV infection at ART initiation (with and without pregnancy) compared to those with late-stage infection, and to explore socio-behavioural factors that (may) influence adherence among the early-stage patients.	Age, social support, disclosure rates, perceived stigma, gender	Predictors of adherence in Uganda and South Africa include, respectively, Increasing age, employment instrumental support, use of Medication other than ART (positive) and Sex work, structural barriers, food insecurity, maladaptive coping, instrumental and cigarette smoking.
74	Masa et al., 2017.[33]	Quantitative Cross-sectional study	Study population (sample n=100)	Zambia: Lundazi district, Eastern Province.	Investigate the role of various components of household economic status in influencing adherence to ART among	Asset ownership (modified by asset type), ownership of transportation-related	The association between household economic security and ART adherence is less straightforward. In particular, the role of economic security on ART

					treatment experienced persons living with HIV (PLHIV) in rural Eastern province, Zambia; and examine alterable economic correlates of ART adherence to help identify factors that can be targeted by adherence interventions.	assets (being busy and always away from home), Place of residence	adherence appears to be a function of the household-level economic variable. While, having more income and having a non-farming related occupation (e.g., construction, trading, or service) were associated with ART adherence, owning land, owning more assets (i.e., mode of transportation and livestock), and food insecurity were associated with ART non-adherence. But these were not statistically significant. Place of residence had a statistically significant effect on adherence (Respondents from Lumezi were less likely to be adherent to ART than respondents from Lundazi).
75	Morojele et al., 2014.[34]	Quantitative	Study Population (sample size n= 304)	South Africa: Tshwane	Examined whether alcohol use is associated with antiretroviral therapy (ART) adherence independently of structural and psychosocial factors among 304 male and female ART recipients in ART sites in Tshwane, South Africa.	alcohol use; structural factors (i.e. food insecurity and time taken to get to the clinic); stigma and HIV non-disclosure	Gender was significantly associated with adherence (with women more likely to be adherent than men). The associations between both employment status and marital status, and ART adherence were marginally significant. Structural factors that were significantly associated with ART adherence include food insecurity, and time to get to the doctor of more than one hour. Also, HIV stigma and HIV non-disclosure affected adherence (negatively).
76	Ramadhani et al., 2007.[35]	Quantitative	150 subjects	Tanzania: Northern Tanzania.	Identify the predictors of incomplete adherence, virologic failure, and antiviral drug resistance in order to facilitate long term ART success.	Paying for one's medication for longer (self-funded treatment), and Disclosure (protective against non-adherence).	Predictors of incomplete adherence on bivariable analysis were sacrificing health care for other necessities (e.g., food, clothing, children's' school fees, or housing; and walking distance to clinic). Disclosure of HIV status to persons other than health care providers and the proportion of months receiving self-funded treatment displayed trends toward significant associations. In multivariable analysis, sacrificing health care for other necessities and the proportion of months receiving self-funded treatment were associated with incomplete adherence, and disclosure of HIV infection status again displayed a trend toward a protective effect against incomplete adherence.
77	Weiser et al., 2012.[36]	Quantitative	Population (n=458 participants). Only (sample n=406) individuals (89%)	Uganda: rural Mbarara District.	Understand the association of food insecurity with morbidity and patterns of healthcare	Food insecurity, hospitalisation, socioeconomic status,	At baseline, moderate and severe food insecurity were negatively associated

			who had no missing data on any variables were included in this analysis.		utilization among individuals accessing ART in rural Uganda.	*Trade-offs between subsistence needs and healthcare needs (competing demands)*	with physical health-related quality of life over follow-up.<br>On competing demands, there was a higher proportion of study participants who gave up food in order to access medical care, and most participants gave up adequate food for themselves and families in order to obtain ART.<br>Food insecure participants had higher likelihood to report giving up ART for food than those who were not food insecure.
78	Weiser et al., 2014.[37]	Quantitative (Longitudinal cohort study)	438 participants	Uganda: rural Mbarara District	Examine the longitudinal associations between food insecurity and HIV treatment response in a cohort of HIV-infected individuals receiving ART in rural Uganda.	Food insecurity	Food insecurity was associated with higher odds of ART non-adherence [Adjusted Odds Ratio [AOR]=1·56, 95% confidence interval [CI]=1·10–2·20; p<0·05], incomplete viral suppression [AOR= 1·52, 95% CI 1·18–1·96; p<0·01], and CD4 cell count <350 [AOR=1·47, 95% CI 1·24–1·74; p<0·01]. Adding adherence as a covariate to the latter two models removed the association between food insecurity and viral suppression, but not between food insecurity and CD4 cell count.
79	Weiser et al., 2017.[38]	Qualitative (Longitudinal study and clinical trial)	54 HIV-infected participants (45 intervention and 9 control) ranging from 23 to 56 years of age	Kenya: Nyanza region.	Understand how and why a livelihood intervention affected the health and health behaviours of HIV-infected Kenyan adults	Food security cum livelihoods interventions as a mechanism for improving ART adherence; addressing structural causes of non-adherence.	Livelihood intervention among PLHIV was linked to improved clinic attendance and ART adherence, hence better health outcomes. Some of the positive mechanisms include: Better food security, improved financial stability, improved productivity, enhancing social support, better control over work situations, and renewed desire (among PLHIV) to prioritise their own health.
80	Murray L et al., 2009.[39]	Qualitative: Two Qualitative data collection methods: Free Listing (FL) and KIIs.	Study participants: Zambia Exclusive Breastfeeding Study (ZEBS) participants. Interviews conducted: 33 KIIs; 92 IDIs (n=125)	Zambia	Explore potentially important factors affecting HIV-infected women's decision to accept or continue with ART	Local Cultural Frameworks, mental and behavioural health, Stigma and motivating factors.	Barriers identified include: Side effects of ART, Hunger, stigma, unfamiliarity with the implications of a chronic, potentially deadly disease, complicated effects of ART on interpersonal relations (e.g. husband/wife), Depression and hopelessness and Lack of accurate Information.
81	Elafros M et al., 2017.[40]	Quantitative	Study population (sample size (n=145))	Zambia: Lusaka	To assess adherence to combination Antiretroviral Therapy (cART) among	Adverse Drug effects	Co-usage of cART and Anti-seizure medication was associated with increased nausea and vomiting after 2

					patients using and those not using Anti-Epilepsy medications concurrently (with ART), as well as to examine the Viral Load (VL) response of a subset of the participants after six months to observe short term and long-term drug adverse effect (impacts) in patients		weeks. In relation to medication adherence in the long term (after 6 months), some participants had decreased medical adherence and often missed clinic visits, raising concerns about the long-term efficacy and tolerability of HIV and Epilepsy co-treatment on resource limited settings.
82	Denison et al., 2015.[41]	Quantitative	A total of 4489 participants were included, of whom 1498 underwent HIV RNA testing.	Tanzania, Uganda and Zambia:<br>18 facilities in the 3 countries	Characterize antiretroviral therapy (ART) adherence across different programmes and examine the relationship between individual and programme characteristics and incomplete adherence among ART clients in sub-Saharan Africa.	Visiting a traditional healer, Alcohol abuse, Medication, stigma	Factors significantly related to incomplete adherence included visiting a traditional healer, screening positive for alcohol abuse, experiencing more HIV symptoms, having an ART regimen without nevirapine and greater levels of internalized stigma.
83	Ehlers V et al., 2015.[42]	Quantitative	300 ART patients between November 2011 and February 2012.	Botswana	Identify factors affecting ART adherence levels and the impact on immunologic and virologic responses in adult patients in one rural district in Botswana and, further, to use this knowledge to enhance the ART adherence rate amongst adult patients at the participating clinic, other clinics in Botswana and other sub-Saharan African (SSA) countries.	Costs incurred through transport and lost wages to visit clinics, forgetfulness, running out of pills, depression. *Side effects and low levels of stigma (Not associated with adherence)*	Motivators of good adherence included disclosure of HIV-positive status to more than one-person, frequent adherence counselling, self-efficacy for adherence to ART, positive interactions between patients and healthcare providers; and using adherence partners. Barriers to adherence were forgetfulness, transportation costs to and from the clinic, time away from work and side-effects.
84	Elul B et al., 2013.[43]	Quantitative	Sample size - 1798 patients; 18 years and above at enrolment; had initiated first line ART at one of the study's sites 6, 12, 18 month [+/-2months] prior to data collection; and still in treatment or transferred into one of the study sites within 30 days of ART initiation and still there	Rwanda: Kigali	To assess determinants of optimal adherence in Kigali, Rwanda	Age, Gender, Treatment (ARV) side-effects, Documentation of CD4 cell count at ART initiation, Alcohol use	A number of patient characteristics were associated with higher odds of non-adherence, including longer time on ART, younger age, experiencing severe side effects, not having a CD4 count at ART initiation, and alcohol use. Patients who had been on ART for 18 months had 75% higher odds of non-adherence compared to those who had been on ART for 6 months.
85	Filimao et al., 2019.[44]	Quantitative (Retrospective) study.	Study population consists of HIV positive individuals in the health facilities 15 years old and older who started ART between January 1,	Mozambique: 3 health facilities of Zambe´zia Province.	Investigating individual level factors associated with non-adherence to ART pick-up in Mozambique.	Age, sex, education level, profession, marital status, residence, health facility, CD4+T cell-	Determinant of non-adherence to ART pick-up include being younger (<35 years), being treated in rural area, taking NVP-based ART regimen, and not participating in CASG.

			2013 and June 30, 2014. Sample size = a total of 1413 patients (included in the analysis).			count, body mass index (BMI), ART-regimen, and whether involved or not in a Community Adherence Support Group (CASG).	
86	Kip E et al., 2009.[45]	Quantitative	Population (sample size n=400)	Botswana	To identify factors influencing patients' anti-retroviral therapy (ART) adherence at four clinics in Botswana	ART Side Effects, Travel expenses, Lack of social support, Use of traditional Medicine, Alcohol abuse, Long clinic waiting times	Patients' and Service centred barriers were central to understanding non-adherence. Patient-centred barriers to ART adherence included inadequate knowledge about ART, human immunodeficiency virus (HIV), acquired immunodeficiency syndrome (AIDS), CD4 cell and viral-load results, stigma, travelling costs, waiting times at clinics, side effects of ART, use of traditional (indigenous or folk) medicines, and abuse of alcohol. Service-centred barriers included nurses' attitudes and knowledge, health workers' inability to conduct home visits and to contact defaulters, limited clinic hours, delays in getting CD4 and viral-load results.
87	Eyassu et al., 2016.[46]	Quantitative	Study population consists of HIV positive adults (18 years and older) who were on ART at the Kwa-Thema clinic. Sample size was 290 (males and females).	South Africa: Gauteng Province	Determine adherence to ART among HIV//AIDS patients	Education level, knowledge of ART benefits, Pills burden, perception of feeling better, ARVs side-effects, Travel/ Migration, being too ill, Economic situation and stigma	Education level, knowledge of ART benefits, Pills Burden, perception of feeling better, ARVs side-effects, Travel/Migration, being too ill, Economic situation and stigma - all these had impacts on adherence to ART
88	Adeniyi et al., 2018.[47]	Mixed methods	Quantitative study population= 1709 participants. The questionnaire was piloted among 20 women who were not included in the study. Qualitative study population= 177 purposively selected HIV infected parturient women, who self-reported non-adherence to ARV.	South Africa: Buffalo/Amathole districts, Eastern Cape Province	Examine adherence levels and factors explaining non-adherence among pregnant women in the Eastern Cape Province in South Africa	Smoking, alcohol use, non-disclosure of HIV status to a family member linked to internalised stigma, education and marital status. Food insecurity, relocation, being busy at work and forgetting (caused by partying and drinking) affect adherence.	Marital status, cigarette smoking, alcohol use and non-disclosure to a family member were the independent quantitative predictors of non-adherence. Women with grade 1–6 level of education were more likely to report complete adherence compared to women who had tertiary level of education.<br>Drug-related side-effects, being away from home, forgetfulness, non-disclosure, stigma and work-related demand were among the main

							qualitative reasons for non-adherence to ART.
89	Mayanja et al., 2013.[48]	Qualitative	Study population 35 patients with poor ART outcomes (drawn from a population of 379 patients eligible from 2004-2011). 5 patients with very poor ART outcomes were qualitatively. The result draws mainly from these interviews.	Uganda	Describe patients' specific personal barriers to ART adherence, health status, CD4 cell counts and viral loads ART outcomes.	Failure to disclose serostatus driven by desires to have children; orphanhood and lack of family support; and adolescence, family instability and sexual desires.	Factors identified from patients' interviews as barriers to (optimal) adherence include: Side effects (headaches, diarrhoea), Lack of treatment supporters, non-disclosure and denial, drug fatigue, age (adolescence), desires to conceive and have sex, reliance on traditional medicine (based on perceived side effects)
90	Aspeling et al., 2008.[49]	Qualitative	11 participants were included in the Qualitative analysis.	South Africa	The objective of the study was to determine factors that influence adherence to ART among HIV-infected black women attending an urban private health-care facility in South Africa.	Religion, fear, social support, traditional medicine/treatment, side-effects, African customs	Factors linked to adherence include medical cost, side-effects, family (parental/partner) support, having children, changing caregivers (interrupts), trusted caregiver (positive), good treatment routine, fear of stigma and discrimination (negative), fear of ART-related myths, culture (household gender-linked economic dynamic), religion.
91	Semvua et al., 2017.[84]	Quantitative	Study population (Analysis sample): n=228 patients enrolled)	Tanzania	Investigate risk factors that predict non-adherence to antiretroviral treatment among HIV-infected individuals in northern Tanzania.	Age, gender, marital status, unemployment	Non-adherence was associated with younger age and Unemployment (based on defining non-adherence as below 95 percent ART collection based on the pharmacy drug refills (PDR) in the last 2 years).
92	Ngarina et al., 2013.[90]	Qualitative	23 HIV-infected women enrolled (CD4 cell count of < 200/μL) who were put on ART for life and followed for 2 years postpartum.	Tanzania: Dar es Salaam	Explore women's own perceived barriers to adherence to ART post-delivery and after the cessation of breastfeeding so as to identify ways to facilitate better drug adherence among women in need of ART for their own health.		Findings show that participants lacked motivation after having succeeded in preventing their child from becoming infected; (2) they did not feel ill; (3) life felt hopeless; (4) living in poverty constrained their ability to adhere; (5) the demands of everyday life were overwhelming; (6) they had to hide their ART due to the stigma of being HIV-infected making it difficult to follow the drug regimen.
93	El-Khatib et al., 2011.[91]	Quantitative	(Analysis sample n=147) A total of 154 women participated in this study. Of these, 147 (95%) had pill count assessment data for at least five out of seven visits during the study period (i.e. The first 24 weeks on	South Africa: Johannesburg (Urban).	Examine socio-economic characteristics associated with incomplete adherence, assessed through pharmacy pill counts, and HIV RNA quantified in plasma among women in a research cohort, from ART initiation through 24 weeks of	Participation in pMTCT program: previous use of single-dose nevirapine (sdNVP) and its impact on adherence-virologic failure nexus within	Two factors strongly linked to incomplete adherence include: level of education lower than (or ending at) grade 11; and lack of financial support from a partner or husband. Other factors like birth outside South Africa, living in informal housing, family member's death (in the first 24 weeks), not having

			ART); data on seven patients (5%) were missing for more than five visits and these were excluded from the final analysis.		treatment (in Johannesburg, South Africa) so as to identify characteristics of women at higher risk of incomplete adherence to better target interventions to enhance adherence and sustain long-term ART success.	NNRTI-containing (ART) regimen; Socioeconomic status: Low education attainment, poor living area (informal settlement), and providing care for many children.	a water source inside the home, low socio-economic status, and reporting a divorce or separation during first 24 weeks on ART also influenced increased viral load (VL >400 copies/ml at 24 weeks) which points to incomplete adherence at 24 weeks. Women reported three main reasons for missing their medication: being away from home, being busy with other things and simply forgetting. The most common reason for missing pills (i.e. being away from home) varied in importance over the first 24 weeks.
94	Lester R et al., 2010.[97]	Quantitative study (multisite randomised Clinical trial): Data from HIV-infected adults initiating ART in 3 clinics.	538 participants were recruited for the study (n=273 belong to the SMS intervention group while the control or standard care was (n=265).	Kenya: Nairobi	Assessing whether mobile phone communication between health-care workers and patients initiating ART in Kenya improved drug adherence and suppression of plasma HIV-1 RNA load.	Mobile phone usage in resource limited settings.	More patients in the SMS Intervention group reported self-adherence above 95 percent than patients in the control group after two visits. After adjusting for baseline covariates, self-reported adherence remained significantly better among patients in the SMS intervention group than the control group. Similarly, patients in the SMS intervention group had suppressed viral loads below the level of detection (less than 400 copies per mL) at 2 months compared to the control group.
95	Randell, H. and Gray, C., 2016.[98]	Quantitative study: Longitudinal survey data matched with high resolution gridded climate data. Survey and stratified random sampling used for household selection.	Study population includes n=3336 individuals (n=1720 boys; n=1616 girls) from 1227 households.	Ethiopia	Examine the intersection between environmental change and education by studying how temperature and precipitation variability impact on schooling outcomes un rural Ethiopia.	Temperature change and precipitation	Early life climatic conditions – namely milder temperatures during all seasons and greater rainfall during the summer agricultural season – are associated with an increased likelihood of a child having completed any education. Females, individuals with older household heads, and individuals for whom the household head has no formal schooling are less likely to have completed any schooling. Additionally, when drought is experienced, the odds of the child completing schooling in early childhood reduces.
96	Peltzer et al., 2012.[100]	Quantitative	A total of 152 adult patients on ART and with adherence problems were randomized	South Africa: UThukela District of KwaZulu-Natal.	Examine whether a lay health worker lead structured group intervention is effective in improving adherence to ART when combined with standard adherence intervention	Group ARV medication intervention training, lay counsellors,	There was a significant improvement of ART adherence and CD4 count and a significant reduction of depression scores over time in both intervention and control conditions; however, no significant intervention effect between intervention and control conditions was

					strategies in a cohort of HIV-infected adults.		found. This means that having a group ARV medication adherence training as well as the control condition (standard care) impacts positively on ART adherence.
97	Molemans et al., 2019.[106]	Quantitative	Sample size of 380 HIV positive patients per survey round (2 Rounds). Analysis sample size (n=699 - control group (n=369), intervention group (n=233), intervention (not followed up) group (n=97).	Eswatini: (Hhohho Region, formerly, Swaziland).	Assess whether the implementation of an 'Early access to ART for all' intervention changed patterns of disclosure, treatment adherence and healthcare interactions.	Early ART initiation, CD4 count, Feeling healthy (vs not), communication with healthcare providers	No changes were found in patterns of disclosure and Treatment Adherence among HIV+ patients in the Early ART initiation groups.
98	Aboubacrine S et al., 2007.[107]	Quantitative	Study participants included a convenience sample of (n=270) HIV patients (176 women and 94 men) treated with ART recruited from May to June 2004, at seven ART-delivery sites in Bamako (n=110) and Ouagadougou (n=160).	Mali and Burkina Faso: 4 hospitals and 3 community-based treatment sites in Bamako (Mali) and Ouagadougou (Burkina Faso).	Determine the prevalence and identify the factors that influence antiretroviral therapy (ART) adherence among patients in Bamako and Ouagadougou.	Having children and a regular partner; and being a housewife (facilitate adherence-link to social support); Treatment-related factors (time on ART; stigma (affecting disclosure and condom-use) – adherence barriers.	In Burkina Faso, individual characteristics were not associated with adherence. However, having children was positively associated with adherence. Length of time on ART was a predictor of adherence. Decline in adherence levels was observed as early as seven months after treatment initiation. In Mali, being a housewife was associated with better adherence. Furthermore, planning to have a child in the next year was associated with non-adherence. Also, time on ART was associated with adherence in both countries with decline in levels of adherence occurring later in Mali.
99	Iwuji et al., 2018.[108]	Quantitative (prospective cohort) study	900 individuals-initiated ART (≥12) months before study end. 71.7 percent were female	South Africa: Hlabisa sub-district in rural KwaZulu-Natal	Study examines ART adherence in a nested cohort study within the ANRS Treatment as Prevention Trial.	Adherence within first 12 months of ART; Out-migration; Gender	Study found no evidence that higher CD4 counts at ART initiation were associated with sub-optimal ART adherence in the first 12 months.
100	Fiorentino et al., 2019.[109]	Quantitative	Study population n=2138 participants (sample n=894 women).	Cameroun	Investigate whether IPV was associated with Antiretroviral therapy interruption (ATI) among WLHIV participating in the ANRS-12288 EVOLCam study survey; and to describe the prevalence of the different forms of IPV in this subpopulation and identify associated risk factors. Study findings focused only on IPV association with recent ATI ⩾ 1m due to the cross-sectional design of the survey.	High IPV (sexual IPV) prevalence;	The risk of recent ATI ⩾ 1m was higher in women suffering from frequent physical IPV than in those reporting NO or occasional IPV. Other factors independently associated with recent ATI ⩾ 1month include experience of ART stock-outs in the previous 3 months, women aged 32–37 years, monthly household income per consumption unit >75th percentile.

101	Moriarty et al., 2018.[110]	Qualitative	Study population: 57 participants sampled from an initial population of 60	Ghana: Kumasi	Describe how patients currently receiving ART react to and cope with stock-outs in order to more thoroughly understand the implications frequent medication stock-outs have for long-term treatment adherence	Health-systems (structural) level factors	Overall, about one-third of participants reported stopping ART at least once due to a medication stock-out. Among those who reported stopping treatment due to a stock-out, several tried to extend their medications by reducing their dosage until they ultimately ran out. Patients developed different strategies to cope with drug stuck-out, some of which were risky in the short and long term.
102	Carlucci et al., 2008.[111]	Quantitative	Study population (n=424 patients receiving ART)	Zambia: The Macha Mission Hospital (MMH)	Investigate the predictors of adherence with a special focus on travel duration and distance as possible predictive factors for non-adherence among rural PLHIV	Individual and community resilience	Travel duration and distance were not predictive of adherence; duration of patients' ART experience
103	Brittain K et al., 2018.[125]	Quantitative	Study population (n=482 women; (median age: 31 years)), enrolled between May 2013 and June 2014.	South Africa: Gugulethu (Cape Town)	Exploring suboptimal adherence to ART, elevated viral load, and factors associated with each of these outcomes among pregnant women already on ART when entering PMTCT services.	Marital status, unintended pregnancy	After adjustment for age, suboptimal adherence was significantly more common among women who were not married/cohabiting and women who reported a higher level of concern about taking ART.
104	Igumbor et al., 2011.[126]	Quantitative (retrospective) study based on patient clinical records.	Study population (n=540 patients records); Female (64%), persons aged between 25 and 39 years old (59%), persons supported by adherence supporters (56%) and patients who lived within a 9 km radius from the health facility (58%).	South Africa	Assess the impact of a community-based adherence support service on the outcomes of patients on Antiretroviral therapy (ART)		A significantly higher proportion of patients with a community-based adherence supporter (also known as a patient advocate, PA) had viral load (VL) of less than 400 copies/ml at six months of treatment (70%, p0·001); a significantly higher proportion of patients with PAs (89%) attained a treatment pickup rate of over 95% (67%; p0·021). Patients at health facilities with PA services maintained a suppressed VL for a longer period as opposed to patients at health facilities without PA services (p0·001), also patients at health facilities with PA services remained in care for longer periods (p0·001). This suggests that integrated community-based adherence support is crucial in ensuring that patients remain in care, regularly pick up their treatment from ART clinics and are virologically suppressed. The study also underscores the importance of access to health services and the

							presence of an enabling environment in the treatment of AIDS
105	Wilson et al., 2016.[127]	Mixed methods (Longitudinal study), including interviews and FGDs.	Study population (n=214 women). Participants were age 18 or older.	Kenya	Evaluate the association between IPV in the past year, detectable viral load and poor ART adherence in HIV-positive female sex workers (FSWs) in Mombasa, Kenya	Resilience, social support, relationship commitment, and non-disclosure of HIV status	Longitudinal analysis of the association between experience of IPV in the past year and ART adherence showed no significant association (even after controlling for age and education) among FSWs. Qualitative data showed that experiencing IPV did not make it more difficult to engage in HIV care, initiate ART, or take their medication (Resilience, social support, relationship commitment, and non-disclosure of HIV status emerged as important factors that may help to explain why IPV was not an important barrier to ART adherence).
106	Avong et al., 2015.[128]	Quantitative	Sample population (n=502)	Nigeria: North Central geopolitical region	Determine the level of adherence to ART and adherence determinants among participants who had been on ART for an average of three and half years.	Age, Forgetfulness	Only age and virologic suppression were significantly associated with adherence to ART. Forgetfulness (43%) was the major reason for non-adherence, while improvement in health condition (40%) was the main facilitator of adherence to the medications.
107	Grimsrud et al., 2016.[129]	Quantitative	Overall population (n=8150 adults initiating ART (2002-2012) followed until the end of 2013. From June 2012, stable patients (on ART >12 months, suppressed viral load) were referred to community-based adherence clubs (CACs). Study sample (n=2113) CAC patients.	South Africa: Gugulethu Community Health Centre (CHC), Cape Town	Describe outcomes [LTFU and viral rebound] over the first 18 months of CAC implementation in Cape Town, South Africa and compares patient outcomes under the CAC model of care to those of patients managed in facility-based primary care.	Age (youth aged 16-24 years), CACs,	Among CAC patients, Loss to follow-up (LTFU) and viral rebound were twice as likely in youth (16–24 years old) compared with older patients, but no difference in the risk of LTFU or viral rebound was observed by sex. CAC participation was associated with a 67% reduction in the risk of LTFU compared with community health centre, and this association persisted when stratified by patient demographic and clinic characteristics.
108	Luque-Fernandez et al., 2013.[130]	Quantitative	Study population (n=2829) individuals were followed.	South Africa: Khayelitsha, Cape Town	Evaluate the effectiveness of adherence clubs compared to traditional clinic-based care in maintaining or improving long-term retention-in-care and virologic suppression	Patients-led adherence clubs and service delivery, Virologic rebound	Club participation was strongly associated with virologic suppression at study entry. At the end of the study, 97% of club patients remained in care compared with 85% of other patients. In adjusted analyses club participation reduced loss-to-care by 57% (hazard ratio [HR] 0·43, 95% CI = 0·21–0·91) and virologic rebound in patients who were initially suppressed by 67% (HR 0·33, 95% CI = 0·16–0·67).

109	Nakamanya et al., 2019.[131]	Qualitative (cross-sectional)	Study sample (n=50). The participants were selected from an initial 953 who had been on ART for six months or more.	Uganda Wakiso and Kalungu	Investigate the role of treatment supporters in sustaining adherence for people living with HIV on long-term ART	Treatment supporters' importance at ART initiation and long term; Gender of treatment support (gender dynamics)	Treatment supporters are important both at ART initiation and long term (mental health and coping support); females more than males were played more supportive roles; and Individuals without treatment supporters seemed to have more challenges than those with continued support.
110	Marconi et al., 2013.[132]	Quantitative	Individuals who are 18 years and above who were receiving more than 5 months of their first ART regimen. Sample size= 158 cases and 300 controls (458 total)	South Africa: Durban	Define individual level determinants/Early Warning Indicators (EWI) of Virologic Failure (VL) for clinicians to use during routine care to compliment WHO population level EWIs.	Age, Gender (MALE), inactive religious faith, family member with HIV, depression, low CD4 count, TV/Radio reminder	Demographic factors (younger age, male); socioeconomic factors (owning a car); psychosocial and behaviour factors (symptoms of depression and fatigue, and practicing unsafe sex, not active in religious faith and having family member with HIV); and clinical factors (Low CD4 count and Having Television/Radio vs Telephonic ART reminders) - all associated with Virologic Failure.
111	Adejumo et al., 2016.[147]	Quantitative	Study participants (n=453).	Nigeria: Ibadan (AIDS Prevention in Nigeria (APIN) clinic, University College Hospital)	Determine association between psychiatric disorder and ART adherence; and whether associations between psychiatric disorders and adherence were mediated by demographic factors such as age, marital status and occupational skill level, or clinical factors such as duration of HIV treatment and CD4 lymphocyte counts.	Depression; ART adherence; increased rates of broad psychopathology in Nigeria; ART Self-report (1-month self-report adherence vs not 1-week self-report (long-term vs short-term adherence self-report))	Within the Participants in Psychiatric group analysis, there was no statistically significant association between the participants' number of mood diagnoses or suicidality symptoms, and their reported number of missed doses in the past week or month; But the odds of having depression were three times higher in participants who were non-adherent in the preceding month, compared to those who were completely adherent (controlling for age, gender, marital status, education, class of occupation, duration of ART use and current CD4 count)

Appendix V: Quality assessment of quantitative and mixed methods studies on ART adherence

	Clear research question with aims/objectives	Appropriate sampling	Study design stated	Data collection method appropriate or prospective	Clarity/completeness in data analysis	Clear presentation of results	Does the discussion/ conclusion support results	Risk of bias
Avong et al 2015	Yes	Yes	Yes	Yes	No	Yes	Yes	At risk
Adejumo O et al 2016	Yes	Yes	No	Yes	No	Unclear	Unclear	At risk
Weiser S et al 2012	Yes	Yes	Yes	No	Yes	Yes	Yes	At risk
Weiser S et al 2014	Yes	Yes	Yes	Yes	Yes	Yes	Yes	Low risk
Semvuna SK et al 2017	Yes	Yes	Yes	No	No	Yes	Yes	At risk
Ramadhani HO et al 2007	Yes	No	Yes	No	Yes	Yes	Yes	At risk
Peltzer K et al 2012	Yes	Yes	Yes	Yes	No	No	Yes	At risk
Molemans M et al 2019	Yes	Yes	Yes	Yes	No	Yes	Yes	At risk
Marconi VC et al 2013	Yes	Yes	Yes	No	Yes	Yes	Yes	At risk
Luque-Fernandez M et al 2013	Yes	Yes	Yes	No	Yes	Yes	Yes	At risk
Grimsrud A et al 2015 (LTFU)	Yes	No	Yes	Yes	Yes	Yes	Yes	At risk
Elul B et al 2013	Yes	No	Yes	Yes	No	Yes	Yes	At risk
Elafros MA et al 2017	Yes	No	No	Yes	No	Yes	Yes	At risk
Ehlers V et al 2015	Yes	Yes	Yes	Yes	No	No	Yes	At risk
Denison JA et al 2015	Yes	No	Yes	No	Yes	Yes	Yes	At risk
Aboubacrine S A et al 2007	Yes	No	Yes	No	No	No	Yes	At risk
Wilson K et al 2016	Yes	Yes	Yes	Yes	Yes	Yes	Yes	Low risk
Morojele NK et al 2014	Yes	No	Yes	Yes	Yes	Yes	Yes	At risk
Kip E et al 2009	Yes	Yes	No	No	Yes	Yes	Yes	At risk
Lester R et al 2010	Yes	Yes	Yes	Yes	Yes	Yes	Yes	Low risk
Igumbor JO et al 2011	Yes	No	Yes	No	No	Yes	Yes	At risk
Eyassu M et at 2016	Yes	No	Yes	No	No	No	Yes	At risk
El-Khatib Z et al 2011	Yes	Yes	Yes	Yes	No	Yes	Yes	At risk
Carlucci JG et al 2008	Yes	Yes	No	No	No	Yes	Yes	At risk
Masa R et al 2017	Yes	No	Yes	Yes	Yes	Yes	Yes	At risk
Haberer JE et al 2019	Yes	Yes	Yes	Yes	Yes	Yes	Yes	Low risk
Filimao DBC et al 2019	Yes	No	Yes	Yes	Yes	Yes	Yes	At risk
Brittain K et al 2018	Yes	No	Yes	Yes	No	Yes	Yes	At risk
Adeniyi OV et al 2018	Yes	Yes	Yes	Yes	No	Yes	Yes	At risk
Fiorentino M et al 2019	Yes	No	Yes	Yes	Yes	Yes	Yes	At risk
Iwuji CC et al 2018	Yes	Yes	Yes	Yes	Yes	Yes	Yes	Low risk

Appendix VI: Quality assessment of qualitative studies on ART adherence

	Researcher role clearly described	Recruitment strategy appropriate to research aims	Data collection method clearly described	Analytic method clearly described	Risk of bias
Weiser S et al 2017	Yes	No	Yes	Yes	At risk
Ngarina M et al 2013	Yes	Yes	Yes	Yes	Low risk
Murray LK et al 2009	Yes	Yes	Yes	No	At risk
Moriarty K et al 2018	No	Yes	Yes	Yes	At risk
Mayanja BN et al 2013	Yes	Yes	Yes	No	At risk
Aspeling HE et al 2008	Yes	Yes	Yes	No	At risk
Nakamanya S et al 2019	No	Yes	Yes	Yes	At risk

Appendix VII: Systems diagram linking drought and ART non-adherence in Africa

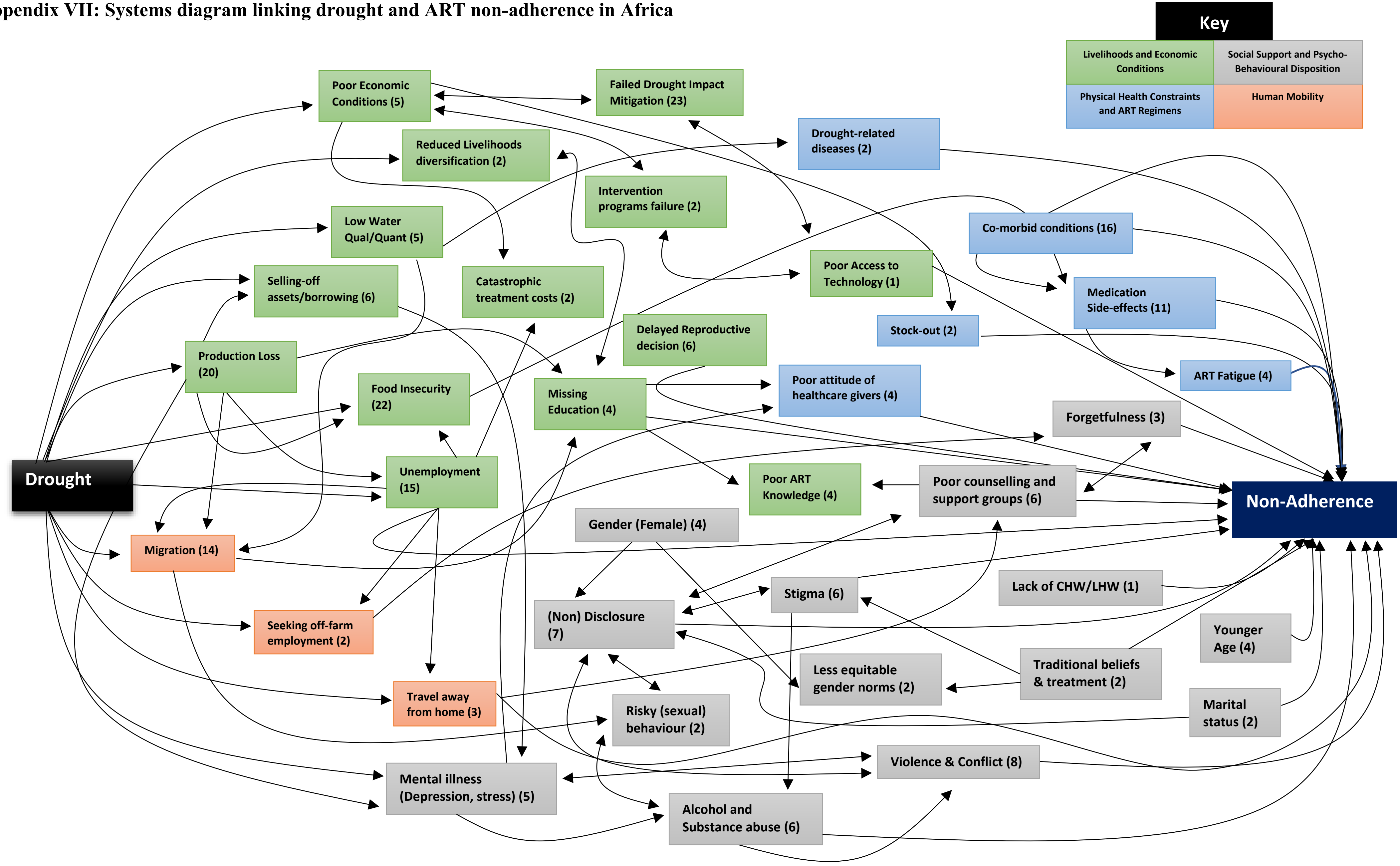

Systems diagram linking drought and ART non-adherence in Africa

This systems diagram demonstrates the complex interlinkages between drought and ART non-adherence moderated by different factors. The colour codes represent different themes (green=Livelihoods and Economic conditions; grey=social support and psycho-behavioural disposition; light blue=Physical health constraints and ART regimens; orange=human mobility). The numbers represent the number of articles referencing a particular factor in this system.

Appendix VIII: PRISMA 2009 Checklist

Section/topic	#	Checklist item	Reported on page #
**TITLE**			
Title	1	Identify the report as a systematic review, meta-analysis, or both.	**1**
**ABSTRACT**			
Structured summary	2	Provide a structured summary including, as applicable: background; objectives; data sources; study eligibility criteria, participants, and interventions; study appraisal and synthesis methods; results; limitations; conclusions and implications of key findings; systematic review registration number.	**1**
**INTRODUCTION**			
Rationale	3	Describe the rationale for the review in the context of what is already known.	**1-2**
Objectives	4	Provide an explicit statement of questions being addressed with reference to participants, interventions, comparisons, outcomes, and study design (PICOS).	**2, Appendix I (p2)**
**METHODS**			
Protocol and registration	5	Indicate if a review protocol exists, if and where it can be accessed (e.g., Web address), and, if available, provide registration information including registration number.	**n/a**
Eligibility criteria	6	Specify study characteristics (e.g., PICOS, length of follow-up) and report characteristics (e.g., years considered, language, publication status) used as criteria for eligibility, giving rationale.	**2, Appendix I (p2)**
Information sources	7	Describe all information sources (e.g., databases with dates of coverage, contact with study authors to identify additional studies) in the search and date last searched.	**2**
Search	8	Present full electronic search strategy for at least one database, including any limits used, such that it could be repeated.	**2**
Study selection	9	State the process for selecting studies (i.e., screening, eligibility, included in systematic review, and, if applicable, included in the meta-analysis).	**2, 3 (Figure)**
Data collection process	10	Describe method of data extraction from reports (e.g., piloted forms, independently, in duplicate) and any processes for obtaining and confirming data from investigators.	**2-3**
Data items	11	List and define all variables for which data were sought (e.g., PICOS, funding sources) and any assumptions and simplifications made.	**n/a**
Risk of bias in individual studies	12	Describe methods used for assessing risk of bias of individual studies (including specification of whether this was done at the study or outcome level), and how this information is to be used in any data synthesis.	**3, Appendix V-VI (pp 33-34)**
Summary measures	13	State the principal summary measures (e.g., risk ratio, difference in means).	**n/a**
Synthesis of results	14	Describe the methods of handling data and combining results of studies, if done, including measures of consistency (e.g., I^2) for each meta-analysis.	**n/a**

Section/topic	#	Checklist item	Reported on page #
Risk of bias across studies	15	Specify any assessment of risk of bias that may affect the cumulative evidence (e.g., publication bias, selective reporting within studies).	**3, Appendix V-VI (pp 33-34)**
Additional analyses	16	Describe methods of additional analyses (e.g., sensitivity or subgroup analyses, meta-regression), if done, indicating which were pre-specified.	**n/a**
**RESULTS**			
Study selection	17	Give numbers of studies screened, assessed for eligibility, and included in the review, with reasons for exclusions at each stage, ideally with a flow diagram.	**3 (Figure)**
Study characteristics	18	For each study, present characteristics for which data were extracted (e.g., study size, PICOS, follow-up period) and provide the citations.	**2-3, Appendix IV (pp 4-32)**
Risk of bias within studies	19	Present data on risk of bias of each study and, if available, any outcome level assessment (see item 12).	**3, Appendix V-VI (pp 33-34)**
Results of individual studies	20	For all outcomes considered (benefits or harms), present, for each study: (a) simple summary data for each intervention group (b) effect estimates and confidence intervals, ideally with a forest plot.	**n/a**
Synthesis of results	21	Present results of each meta-analysis done, including confidence intervals and measures of consistency.	**3-7**
Risk of bias across studies	22	Present results of any assessment of risk of bias across studies (see Item 15).	**3, Appendix V-VI (pp 33-34)**
Additional analysis	23	Give results of additional analyses, if done (e.g., sensitivity or subgroup analyses, meta-regression [see Item 16]).	**n/a**
**DISCUSSION**			
Summary of evidence	24	Summarize the main findings including the strength of evidence for each main outcome; consider their relevance to key groups (e.g., healthcare providers, users, and policy makers).	**7-8**
Limitations	25	Discuss limitations at study and outcome level (e.g., risk of bias), and at review-level (e.g., incomplete retrieval of identified research, reporting bias).	**8**
Conclusions	26	Provide a general interpretation of the results in the context of other evidence, and implications for future research.	**7-8**
**FUNDING**			
Funding	27	Describe sources of funding for the systematic review and other support (e.g., supply of data); role of funders for the systematic review.	**8**

From: Moher D, Liberati A, Tetzlaff J, Altman DG, The PRISMA Group (2009). Preferred Reporting Items for Systematic Reviews and Meta-Analyses: The PRISMA Statement. PLoS Med 6(7): e1000097. doi:10.1371/journal.pmed1000097

For more information, visit: **www.prisma-statement.org**.

Page numbers refer to the PDF version of the paper.
